# Potentially inappropriate prescribing for adults living with diabetes mellitus: a scoping review

**DOI:** 10.1007/s11096-022-01414-7

**Published:** 2022-07-01

**Authors:** Mohammed Biset Ayalew, M. Joy Spark, Frances Quirk, Gudrun Dieberg

**Affiliations:** 1 Pharmacy, School of Rural Medicine, University of New England, Armidale, NSW 2351 Australia; 2 Department of Clinical Pharmacy, School of Pharmacy, University of Gondar, Gondar, Ethiopia; 3 School of Rural Medicine, University of New England, Armidale, NSW 2351 Australia; 4 Biomedical Science, School of Science and Technology, University of New England, Armidale, NSW 2351 Australia

**Keywords:** Inappropriate prescribing, Contraindications, Omissions, Diabetes mellitus, Scoping review

## Abstract

**Background:**

People living with diabetes often experience multiple morbidity and polypharmacy, increasing their risk of potentially inappropriate prescribing. Inappropriate prescribing is associated with poorer health outcomes.

**Aim:**

The aim of this scoping review was to explore and map studies conducted on potentially inappropriate prescribing among adults living with diabetes and to identify gaps regarding identification and assessment of potentially inappropriate prescribing in this group.

**Method:**

Studies that reported any type of potentially inappropriate prescribing were included. Studies conducted on people aged < 18 years or with a diagnosis of gestational diabetes or prediabetes were excluded. No restrictions to language, study design, publication status, geographic area, or clinical setting were applied in selecting the studies. Articles were systematically searched from 11 databases.

**Results:**

Of the 190 included studies, the majority (63.7%) were conducted in high-income countries. None of the studies used an explicit tool specifically designed to identify potentially inappropriate prescribing among people with diabetes. The most frequently studied potentially inappropriate prescribing in high-income countries was contraindication while in low- and middle-income countries prescribing omission was the most common. Software and websites were mostly used for identifying drug-drug interactions. The specific events and conditions that were considered as inappropriate were inconsistent across studies.

**Conclusion:**

Contraindications, prescribing omissions and dosing problems were the most commonly studied types of potentially inappropriate prescribing. Prescribers should carefully consider the individual prescribing recommendations of medications. Future studies focusing on the development of explicit tools to identify potentially inappropriate prescribing for adults living with diabetes are needed.

**Supplementary information:**

The online version contains supplementary material available at 10.1007/s11096-022-01414-7.

## Impact statements

- More studies that assess potentially inappropriate prescribing in low- and middle-income countries, nursing homes and community dwelling settings are needed.
- Explicit tools specifically designed to identify potentially inappropriate prescribing for adults living with diabetes are lacking.

## Introduction

Inappropriate prescribing is a potential threat to patient safety [1]. It may be manifested in the form of overprescribing, misprescribing or underprescribing [2, 3]. Overprescribing is the prescription of a medication that has no clear indication or prescribing two or more agents for the same purpose while a single agent is sufficient. Misprescribing includes prescribing medications at the wrong dose, duration, or frequency, not choosing the first line option or prescribing medications with significant interactions. Underprescribing involves the omission of a beneficial medication [4].

Inappropriate prescribing may result in adverse drug reactions, hospitalization, worsening of the disease and death.[4] People with two or more potentially inappropriate prescribing (PIP) indicators have been reported to have twice the risk of emergency visits and adverse events [5]. Nearly one-third of adverse drug reactions in a Swedish older population were attributed to PIP [6]. In addition to increased risk of morbidity and mortality, PIP can also increase health care utilization. A study conducted in Northern Ireland in 2010 estimated that the annual gross cost of PIP was more than €6 million [7].

The two most common factors that contribute to PIP are polypharmacy (5 or more medications) and multiple morbidity (MM) [8, 9]. One of the high-risk patient groups for polypharmacy and MM are people with diabetes mellitus (DM). The prevalence of DM is increasing globally [10]. The International Diabetes Federation (IDF) reported that 463 million people had diabetes in the year 2019 and this is expected to increase to 700 million by 2045 [11]. Diabetes costs the world more than a trillion dollars currently and is estimated to cost US$2.1 trillion by the year 2030 [12]. Globally, about 5 million deaths per year are due to DM [13]. Most people with DM have MM or complications that can include hypertension, dyslipidaemia, nephropathy, neuropathy, retinopathy, cardiovascular, cerebrovascular or peripheral vascular diseases and as a result receive multiple medications. Correspondingly, PIP among people with DM has been reported to be high in a range of international studies [14–18].

Studies have focused on one or more types of PIP (unnecessary drug therapy, omission, contraindication, inappropriate drug selection (IDS), dosing problems, and drug-drug interactions (DDI)) often utilizing different tools for the same type of PIPs. However, no published review article mapping studies on all types of PIP among people with DM was found in our preliminary search on the Cochrane Database of Systematic Reviews, PubMed, JBI Database of Systematic Reviews and Implementation Reports, PROSPERO, Scopus, Informit, ProQuest, EBSCO and Google scholar databases.

### Aim

This scoping review aims to explore and map the available evidence and to identify gaps regarding identification and assessment of PIP for adults living with DM.

## Method

This scoping review followed the Joanna Briggs Institute (JBI) guidelines for scoping reviews[19] and the published protocol [20].

### Study selection

#### Participants

Studies conducted on adults with DM were included in this review. Studies conducted on people aged < 18 years or adults with a diagnosis of gestational DM or prediabetes were excluded.

#### Concept

PIP is the concept of interest for this scoping review. Studies that reported any type of PIP including contraindications, omissions, dosing problems, DDI, IDS or unnecessary medications were included.

#### Context

Studies conducted in any health care facility (e.g. hospitals, nursing homes) or clinical setting (e.g. inpatient, outpatient) with no restriction to region, country or geographic area were considered for this review.

#### Types of sources

Studies with any type of research design were considered for this review. Various types of existing published and unpublished literature including primary research articles, reviews, case reports, theses and dissertations were included. No language restrictions were applied.

### Search strategy

The strategy for literature searching was developed with the assistance of a librarian at the University of New England. A three-step search strategy, as suggested by JBI Reviewer’s Manual, was utilized [19]. The first step was an initial limited search on two databases (PubMed and ProQuest). The words in the title and abstracts of the identified studies from these two databases were analysed to create search terms for the subsequent step. In addition, MeSH terms, keywords and thesauruses were searched for the key concepts of inappropriate prescribing and diabetes. The second step applied the full search to multiple databases using the index terms and key words identified in the first step. The following databases were searched from their inception to December 2020: PubMed, EBSCO, Web of Science, ProQuest, Scopus, Cochrane, EMBASE, and Informit. In addition, some grey literature sources including Open Dissertation.org, Open Access Theses and Dissertation, and BIELEFELD Academic Search Engine (BASE) were searched for any unpublished work. In a third step, manual searches of reference lists of included studies were searched for additional articles. The final search results were exported to EndNote X8.2 (Clarivate Analytics, PA, USA) and duplicates were removed. A sample search result on PubMed database is shown in the electronic supplementary material (Supplementary Table 1).

### Extraction of results

Data extraction was performed using Colandr (Science for Nature and People Partnership, Conservation International, and DataKind, CA, USA), an online application for conducting systematic synthesis of evidence. Data extracted from the studies included author, year, country, publication type (e.g. original article), study design, study population, setting, sample size, types of PIP investigated, prevalence of PIP, medications involved, examples of PIP events, and criteria used in the identification of PIP. Pretesting of the data extraction form was done on a random sample of 10 articles and modified to ensure that all the required information was captured. One study team member (MB) extracted the data and this was manually checked and verified by others (GD, JS).

### Data synthesis

Data was exported to Excel then to IBM SPSS version 25 for descriptive statistical analysis. Some of the variables (e.g. year of publication, study country) were categorized into groups. Countries were categorized as high-income and low- and middle-income based on 2020–2021 World Bank classification [21]. The mapping of the included studies was undertaken in terms of the type of PIPs studied against study area, setting, year of publication, and criteria used to assess PIP. All DM related PIPs reported from each study were listed and presented in tables. Prevalence of PIP was summarized using median and interquartile range (IQR).

## Results

### Study selection

**Fig. 1:**
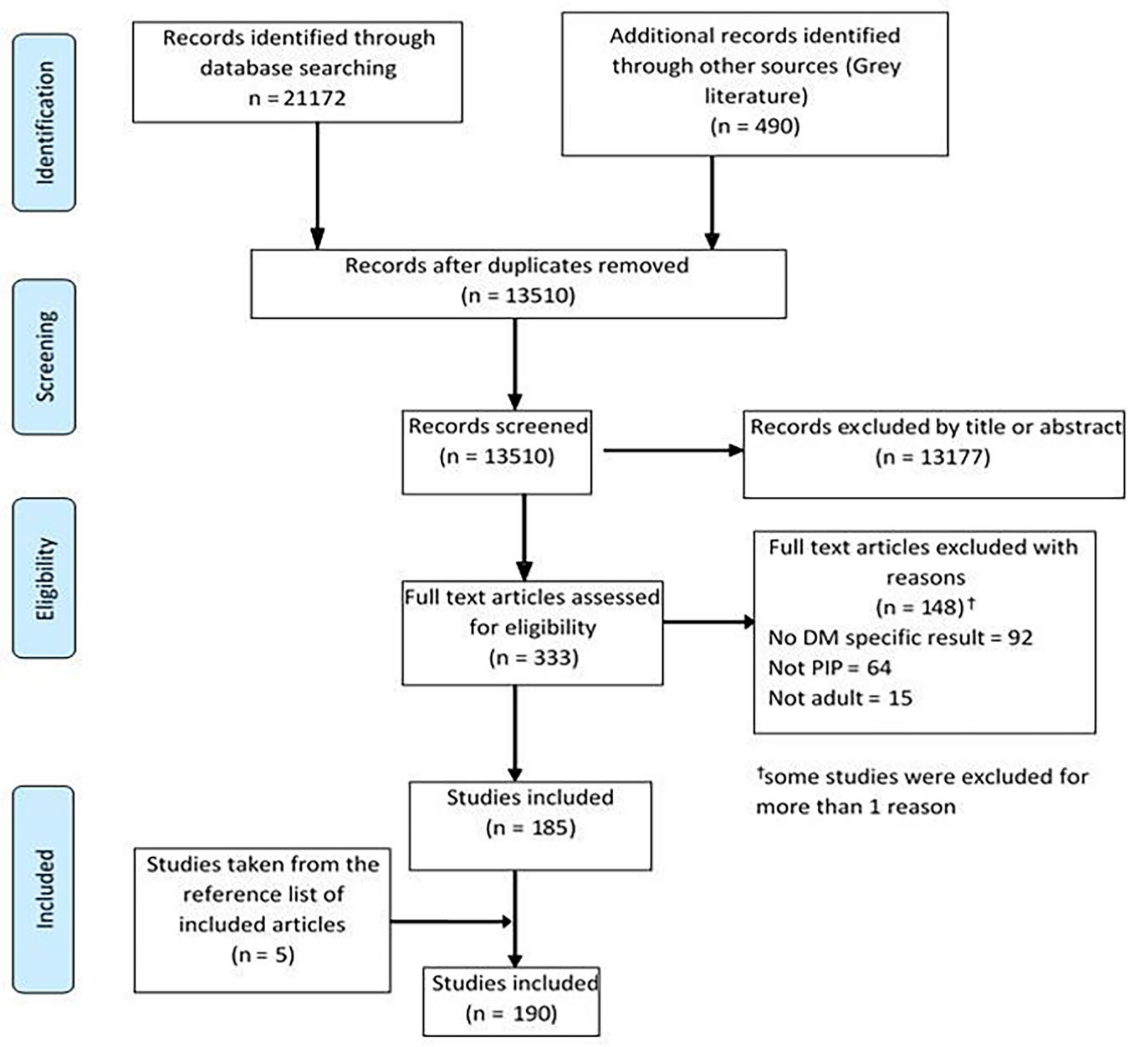
Preferred Reporting Items for Systematic reviews and Meta-Analyses extension for Scoping Reviews flow diagram

As shown in the PRISMA extension for scoping review (PRISMA-ScR) flow diagram (Fig. 1), the systematic search resulted in 21,172 published articles and 490 items of grey literature. After removing duplicates, screening the titles, abstracts and full text and adding articles from the reference lists of included studies a total of 190 articles were found eligible for this scoping review.

### Study characteristics

Of the 190 included studies, 75.8% were published in the last 10 years. The majority (63.7%) of studies were conducted in high-income countries. Methodologically, 74.7% had a cross-sectional nature (Table 1). Detailed characteristics of included studies are provided in the electronic supplementary material (see Supplementary Table 2).

**Table 1:** Characteristics of included studies

Variable	Category	Number (%)
**Year of publication**	Before 2000	6 (3.2%)
	2000–2009	40 (21.1%)
	2010 and after	144 (75.8%)
**Article type**	Original article	173 (91.1%)
	Case report	8 (4.2%)
	Review	2 (1.1%)
	Thesis	7 (3.7%)
**Language of publication**	English	174 (91.6%)
	Non-English	16 (8.4%)
**Study area**	High-income countries	121 (63.7%)
	Low- and middle-income countries	68 (35.8%)
**Study methodology**	Cross-sectional	142 (74.7%)
	Cohort	28 (14.7%)
	Interventional	16 (8.4%)
	Not reported	4 (2.1%)
**Study setting**	Outpatient	100 (52.6%)
	Inpatient	70 (36.8%)
	Community dwelling	17 (8.9%)
	Nursing home	8 (4.2%)
	Not reported	10 (5.3%)

### Criteria for assessment of PIP

Appropriateness of medication therapy was assessed by using a variety of standard references and criteria across the studies. These criteria are summarized in Table 2.

**Table 2:** Types of potentially inappropriate prescribing and standard references/criteria used to assess potentially inappropriate prescribing

Types of PIP and Standard References/Criteria		Number of Studies (%)^a^
**Types of PIP**	Contraindication (CI)	91 (47.9%)
	Prescribing omission (PO)	78 (41.1%)
	Dosing problem (DP)	65 (34.2%)
	Drug-drug interaction (DDI)	56 (29.5%)
	Inappropriate drug selection (IDS)	41 (21.6%)
	Unnecessary drug therapy (UDT)	37 (19.5%)
**Standard References/** **Criteria**	Clinical practice guidelines	66 (34.7%)
	ADA guideline	19 (10.0%)
	Malaysian clinical practice guideline	6 (3.2%)
	NICE guideline	5 (2.6%)
	Canadian clinical practice guideline	3 (1.6%)
	Others	44 (23.2%)
	Explicitly listed criteria (tools)	51 (26.8%)
	STOPP Criteria	22 (11.6%)
	START Criteria	18 (9.5%)
	Beers criteria	22 (11.6%)
	Medication Assessment Tool (MAT)	2 (1.1%)
	Others^b^	8 (4.2%)
	Medication/disease information software and websites	31 (16.3%)
	Micromedex	18 (9.5%)
	Medscape	6 (3.2%)
	Drugs.com	3 (1.6%)
	Lexicomp	3 (1.6%)
	Others^c^	6 (3.2%)
	Summary of medicinal product characteristics (SMPC)	23 (12.1%)
	Books	14 (7.4%)
	Not reported	34 (17.9%)

^a^Some studies used more than 1 criteria

^b^Tool to Reduce Inappropriate Medications (TRIM); Assessing Care of Vulnerable Elders-3 (ACOVE-3) Tool; McLeod Criteria; Mast et al. tool (unpublished); van Roozendaal BW and Krass I checklist for DRP in T2DM; PRescribing Optimally in Middle-aged People’s Treatments (PROMPT) criteria; prescribing quality indicator (PQI)

^c^Pharma software, CheckTheMeds, Swedish Finnish INteraction X-referencing (SFINX) database, I fact software, drug interaction module of the German Bundesvereinigung Deutscher Apothekerverbände (ABDA) database, Healthcare Effectiveness Data and Information Set (HEDIS)

**Abbreviations**: ADA = American Diabetes Association; NICE = National Institute for Health and Care Excellence; START = Screening Tool to Alert to Right Treatment; STOPP = Screening Tool of Older Persons’ Prescriptions

### Types of PIP studied

PIPs reported in the included studies were grouped into 6 major classes. Nearly half (47.9%) of the studies reported contraindications. Prescribing omissions were reported by 41.1% of the studies. Unnecessary drug therapy was the least frequently reported PIP (Table 2).

**Fig. 2:**
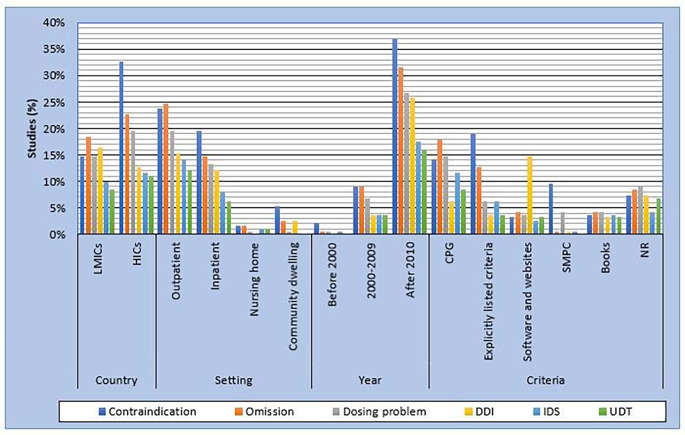
Distribution of studies on different types of potentially inappropriate prescribing by country, setting, year of publication and criteria for assessing potentially inappropriate prescribing. **Abbreviations**:?CPG = clinical practice guideline; DDI = drug-drug interaction; HICs = high-income countries; IDS = inappropriate drug selection; LMICs = low- and middle-income countries; NR = not reported; SMPC = summary of medicinal product characteristics; UDT = unnecessary drug therapy

The most frequently studied type of PIP in high-income countries (HICs) was contraindication, while in low- and middle-income countries (LMICs) prescribing omissions were more common. Prescribing omissions were more frequently studied in the outpatient setting, whereas contraindication dominated in inpatients and community dwelling patients. The use of software and websites as reference/criteria was more frequently reported in studies that identified DDIs, while clinical practice guidelines (CPG) and explicitly listed criteria were common in studies that identified prescribing omissions and contraindications, respectively (Fig. 2).

### Specific PIP events and medications involved

The most common PIP events were contraindications – prescribing metformin in the presence of contraindications (e.g. renal failure) and prescribing medications listed in the Beers Criteria or STOPP list for older adults. Not prescribing antiplatelet and lipid lowering agents for eligible individuals were the most commonly reported prescribing omissions. Prescribing incorrect insulin or metformin doses, not adapting the dose of a medication for renal insufficiency and not keeping to the maximum daily dose recommendations were some of the dosing problems reported in the reviewed studies. Examples of specific events in each class of PIP are shown in Table 3. All PIP events reported in the included studies and medications involved are summarized in the electronic supplementary material (see Supplementary Table 3).

**Table 3:** Examples of specific potentially inappropriate prescribing events and medications involved

Type of PIP	Specific PIP events (examples)
**Contra** **indication**	**Prescribing –** • metformin for a patient with elevated SCr concentration (e.g. SCr > 0.132 mmol/L, SCr ≥ 1.4 mg/dL for women and ≥ 1.5 mg/dL for men, eGFR < 30 mL/min/1.73 m^2^, AKI/CKD, GFR < 45 mL/min, GFR < 60 mL/min/1.73 m^2^), lactic acidosis, pH < 7.35, DKA, use of contrast dye, acute MI, cardiac failure, IHD, CAD, hepatic impairment, dehydration, alcoholism (acute or chronic), respiratory failure, gangrene, pancreatitis, circulatory collapse, stress, metabolic diseases, undergoing surgery, age > 80 years, peripheral vascular disease or proteinuria • sulphonylureas for an older adult (e.g. aged ≥ 75 years), history of HF, unstable angina, CHD, stroke, MI, chronic renal insufficiency (moderate to severe), CKD stage ≥ 3b, history of severe hypoglycaemia, obesity, cognitive impairment, and risky occupation (bus/taxi/train driver, working at height), history of DKA, metabolic acidosis, treatment with bosentan or severe hepatic impairment • glyburide for a patient with CrCl < 50 mL/min, eGFR < 60 mL/min/1.73 m^2^, or frequent occurrence of hypoglycaemic episodes • a long-acting sulfonylurea (e.g. glyburide/glibenclamide, glimepiride, chlorpropamide) for an older T2DM patient (age > 65, age > 45) • insulin aspart, lispro or regular insulin for an older diabetic patient (age ≥ 65) • β-blockers in a diabetic patient with frequent episodes of hypoglycaemia (≥ 1 episode per month), chronic airways disease, taking oral hypoglycaemics or insulin, or older and frail DM patient • metformin for a patient aged 85 years old or above • TZDs in a patient with moderate to severe HF, in AHA class III or IV CHF, liver failure or without investigation of its function, < 18 years old, history of T1DM, concomitantly with metformin in the presence of renal inefficiency, or pregnancy • α-1 blockers, amiodarone, short-acting or immediate release nifedipine, glimepiride, amitriptyline or drugs categorized as high risk in Beers criteria for an older patient • ACEIs for a patient with ESRD • spironolactone for a patient with eGFR < 30 mL/min • aspirin for a patient with CrCl < 10 mL/min • pregabalin fora patient with dizziness, angioedema, decreased platelet count, or non-epileptic seizures • duloxetine for a patient with uncontrolled hypertension, severe renal disease, slow gastric emptying, hyponatremia, urinary hesitation and/or retention, hepatic insufficiency, bipolar disorder, alcohol use, moderate-high severity skin reaction, narrow-angle glaucoma, or non-epileptic seizures • insulin for a patient with BG < 3.9 mmol/l or hypoglycaemia • DPP-4 inhibitors for a patient with hypoglycaemia and concomitant use of insulin or sulphonylurea and pancreatitis • biguanides (e.g. phenformin, metformin) for a high-risk patient for lactic acidosis (e.g. renal insufficiency) • GLP-1 agonist in CKD stage ≥ 4 • gliflozins (SGLT2 inhibitors) for a patient with GFR < 45 mL/min • gliclazide for patient with renal impairment • pioglitazone for a patient having osteoporosis
**Prescribing omission**	**Not prescribing –** • antiplatelets (e.g. aspirin, clopidogrel) for an eligible patient (adult with major cardiovascular risk factors, history of CVD, HTN, hypercholesterolemia, smoking history, TIA/stroke, age ≥ 30 years, or ≥ 40 years, history of atherosclerotic cardiovascular disease in a patient with sinus rhythm, CHD or high risk for CHD, macrovascular disease, IHD, PVD, nephropathy, or microalbuminuria) • lipid lowering therapy (e.g. statin) for an eligible patient (adult with high cardiovascular risk, age > 40 years, history of CVD where the patient’s functional status remains independent for daily activities and life expectancy is more than 5 years, CKD, diabetes duration longer than ten years, HTN, cigarette smoker, family history of early CAD, or albuminuria) • ACEIs/ARBs for an adult with uncontrolled blood pressure, diabetic nephropathy, albuminuria (> 30 mg/24 h), chronic HF, MI, HTN with a history of HF, left ventricular hypertrophy, IHD, CKD, or cardiovascular accident • metformin for an overweight T2DM patient, a T2DM patient ± metabolic syndrome • antihypertensive therapy for hypertensive patient • β-blockers for a T2DM patient with MI, CHF, or HTN + IHD • dual antihypertensive agent for a stage II hypertensive patient • insulin after hypoglycaemia • tricyclic antidepressant for a patient with diabetic neuropathy • fibrates for an adult with TG > 4.5 mmol/L
**Dosing problem**	**Prescribing –** • incorrect insulin dose (high dose, low dose, dose not adjusted when BG persistently > 14 mmol or < 4 mmol, incorrect sliding scale, or sliding scale for an older patient) • incorrect metformin dose (high dose, incorrect dose and interval, thrice daily dosing for SR preparation, dose not adjusted for renal failure, above 1500 mg/d in CKD stage 3a, above 1000 mg in CKD stage 3b, or > 2.5 g/day for older an adult) • unadjusted dose for renal function (unadjusted dose of oral antidiabetic drugs, hypoglycaemic sulfamide, DPP-4 inhibitors, sitagliptin, simvastatin, furosemide, or statin) • high dose digoxin for an older adult (≥ 0.125 mg/d except for treating atrial arrhythmias) • glibenclamide with incorrect dose and interval • unadjusted dose of oral antidiabetic drugs when blood glucose is > 14 mmol or < 4 mmol persistently • excessive dose of sitagliptin • aspirin > 150 mg/day for an older adult • low dose carvedilol for dilated cardiomyopathy • simvastatin at more than 20 mg while receiving amlodipine • without considering dosage reduction for older adults
**Drug-drug interaction**	• aspirin + lisinopril/enalapril/glibenclamide/insulin/clopidogrel/coumadin/ACEIs/ enoxaparin/ketorolac/enoxaparin/lornoxicam/diclofenac/piroxicam/heparin/ glimepiride/NSAID/simvastatin/furosemide/SSRI/metimazole/warfarin/glipizide • metformin + ciprofloxacin/cimetidine/atenolol/enalapril/carvedilol/ranitidine/ salbutamol/furosemide/clarithromycin/spironolactone/levothyroxine/moxifloxacin/ nifedipine/aspirin/captopril/HCT/simvastatin/paracetamol/budesonide • insulin + metformin/aspirin/ciprofloxacin/moxifloxacin/bisoprolol/losartan/ enalapril/carvedilol/captopril/atenolol/thiazide/timolol/levofloxacin/metoprolol/ ACEIs • simvastatin + macrolide antibiotics/ketoconazole/itraconazole/amlodipine/ fenofibrate/warfarin/amlodipine/diltiazem/phenofibrate/verapamil • glimepiride + aspirin/salbutamol/metoprolol/fluconazole/ramipril/sitagliptin/ bisoprolol/ibuprofen/furosemide/losartan/ciprofloxacine/budesonide/warfarin/ lisinopril • atorvastatin + macrolide antibiotics/ketoconazole/itraconazole/macrolide antibiotic/ simvastatin/rosuvastatin/clopidogrel/sitagliptin • duloxetine + metoclopramide/aspirin/ciprofloxacin/anticoagulants/antiplatelet drugs/ NSAIDs/tramadol/metoclopramide • glibenclamide + diclofenac/ranitidine/hydrocortisone/simvastatin/antacid/ topiramate • digoxin + HCT/verapamil/amiodarone/warfarin/furosemide/atorvastatin/ spironolactone • atenolol + amlodipine/gliclazide/glibenclamide/verapamil/carvedilol/clonidine • amiodarone + amlodipine/atenolol/amitriptyline/fluoxetine/digoxin/nepheline • fluoxetine + amitriptyline/haloperidol/diclofenac • glipizide + warfarin/ciprofloxacin/enalapril • pregabalin + naproxen/enalapril/captopril • lisinopril + furosemide/KCl • pioglitazone + insulin glargine/ciprofloxacin • sulfonylureas + trimethoprim/sulfamethoxazole/ACEIs/CYP2C9-inhibitors/anti-hyperlipidaemic • antidiabetics + diuretics/ACEI/anti-lipidaemic drugs/corticoids/other drugs that have a hypoglycaemic effect/β-blockers • CCBs + β-blockers/clopidogrel • thiazide diuretic + ACEIs/ARBs + NSAIDs • NSAIDs + ACEIs/ARBs/β-blockers/spironolactone; ACEIs + ARBs • losartan + spironolactone; pravastatin + darunavir; repaglinide + brotizolam; enalapril + losartan; furosemide + gentamicin; nifedipine + erythromycin; HCT + carbamazepine
**Inappropriate drug selection**	**Prescribing –** • antidiabetic other than metformin as an initial therapy for T2DM • only glibenclamide for an obese DM patient • combined oral therapy without starting with monotherapy • insulin for a patient who need tablet treatment • incorrect insulin type, insulin glargine instead of insulin detemir • monotherapy with long-acting insulin, rapid-acting insulin, or GLP-1 agonist • improper combination of short-, intermediate-, or long-acting insulin • non-statin therapy in a statin-eligible patient • inappropriate intensity statin/low-intensity statin in a high CVD risk patient • non-recommended dual therapy, or triple therapy (add-on therapy to insulin, insulin + metformin, or α-blockers such as prazosin and doxazosin as second or third add-on therapies when other better alternatives were available and not contraindicated) • unadjusted antidiabetic drug while HbA1c value is higher or lower than the patient’s target range • spironolactone and furosemide while thiazide-like diuretic is preferred • CCBs (e.g. amlodipine) instead of ACEIs/ARBs for HTN treatment • drug was fragmented despite being a special oral formulation
**Unnecessary drug therapy**	**Prescribing –** • antiplatelet (e.g. aspirin) or statin for illegible individuals • intensified antidiabetic medication for a patient with limited life expectancy or already at goal HbA1c • dual antihypertensive agents for a stage I hypertensive patient • loop diuretics in the absence of clinical signs of HF • aspirin to a patient with cardiovascular and/or coronary risk < 1.0 event/100 patients/year

**Abbreviations**: ACEI = angiotensin converting enzyme inhibitor; AHA = American Heart Association; AKI = acute kidney injury; ARBs = angiotensin II receptor blockers; BG = blood glucose; CAD = coronary arterial disease; CCB = calcium channel blocker; CHD = coronary heart disease; CKD = chronic kidney disease; CrCl = creatinine clearance; CVD = cardiovascular disease; Cyp2C9 = cytochrome 2C9; DKA = diabetic ketoacidosis; DM = diabetes mellitus; DPP = dipeptidyl peptidase; eGFR = estimated glomerular filtration rate; ESRD = end stage renal disease; GFR = glomerular filtration rate; GLP = glucagon-like peptide; HbA1c = haemoglobin A1c; HCT = hydrochlorothiazide; HF = heart failure; HTN = hypertension; IHD = ischaemic heart disease; KCl = potassium chloride; MI = myocardial infarction; NSAIDs = non-steroidal anti-inflammatory drugs; PVD = peripheral vascular disease; SCr = serum creatinine; SGLT = sodium-glucose co-transporter; SR = sustained release; SSRI = selective serotonin reuptake inhibitor; T1DM = type 1 diabetes mellitus; T2DM = type 2 diabetes mellitus; TG = triglyceride; TIA = transient ischaemic attack; TZD = thiazolidinedione

### Prevalence of potentially inappropriate prescribing

Prevalence of PIP was reported from the included studies in two different ways. Some of the studies reported prevalence for the number of adults with DM in the sample and others took the total number of drug related problems (DRPs). The reported prevalence for each type of PIP varies greatly across the studies (Table 4).

**Table 4:** Prevalence of potentially inappropriate prescribing

Types of PIP	Percentage calculated from	Number of studies reported	Range of reported prevalence	Median prevalence	IQR
**Contraindication**	Adults with DM	41	1.0 − 93.4%	21.6%	8.0 − 34.9%
	DRPs identified	9	0.3 − 7.5%	1.4%	0.8 − 4.3%
**Dosing problem**	Adults with DM	26	2.4 − 63.0%	12.5%	6.2 − 27.8%
	DRPs identified	19	4.0 − 49.3%	17.9%	12.7 − 37.8%
**Drug-drug interaction**	Adults with DM	24	4.0 − 96.0%	45.1%	17.4 − 61.4%
	DRPs identified	12	0.4 − 18.2%	9.1%	1.0 − 17.6%
**Inappropriate drug selection**	Adults with DM	13	3.1 − 90.6%	13.3%	8.0 − 37.0%
	DRPs identified	16	1.0 − 37.0%	10.7%	3.3 − 21.7%
**Prescribing omission**	Adults with DM	29	2.9 − 91.2%	26.7%	16.3 − 54.9%
	DRPs identified	21	2.0 − 49.3%	19.1%	9.7 − 26.2%
**Unnecessary drug therapy**	Adults with DM	10	1.0 − 43.0%	14.1%	2.7 − 27.9%
	DRPs identified	18	0.7 − 29.7%	8.8%	4.0 − 17.2%

**Abbreviations**: DM = Diabetes Mellitus; DRPs = Drug Related Problems; IQR = Inter Quartile Range

### Identified gaps regarding PIP for adults with DM

The following gaps were identified in the study of PIP for adults with DM.

- PIP is less studied in low- and middle-income countries (LMICs) where the risk of PIP could be high due to less efficient systems and resource scarcities.
- PIP is less studied in nursing home and community dwelling adults living with DM.
- The specific events and conditions that were considered as inappropriate prescribing were inconsistent across included studies.
- There are no explicit tools/criteria solely designed to identify PIP among adults with DM.

## Discussion

The current scoping review included 190 studies reporting on PIP in adults living with DM worldwide. To the authors’ knowledge, this review is the first to comprehensively map, identify and assess PIP in this group. The extent and degree of identification of PIP varied with the type of tool/criteria used. Even though explicit criteria are less costly to use than implicit criteria, require less clinical judgement, and can ensure a higher degree of objectivity, only a quarter of the included studies used explicit criteria. Moreover, almost all the explicit tools used were not specifically designed to detect PIP among people with DM. The majority of explicit tools were designed to identify omissions and contraindications in older populations (e.g. Beers criteria, STOPP and START criteria).

### Management of hyperglycaemia

Metformin is the preferred first line pharmacologic agent for people with T2DM, unless contraindicated, because metformin is safe, effective, inexpensive and has beneficial effects on HbA1c, weight loss, and cardiovascular mortality [22, 23]. Many of the reviewed studies considered omission of metformin for adults diagnosed with T2DM as PIP [24–34].

Sulphonylureas should be used with caution because of their risk of serious hypoglycaemia [35]. If the use of sulphonylureas in older individuals is the only option, short acting sulphonylureas (e.g. glipizide) are preferred. Glyburide, a longer acting sulphonylurea, should not be used in older adults because of the increased risk of hypoglycaemia in this patient group [36]. In line with this, many studies that investigated inappropriate prescribing reported that glyburide or chlorpropamide use in an older adult with DM was contraindicated [24, 26, 27, 30–32, 34, 37–43].

Thiazolidinediones (TZDs) used in the presence of contraindication was reported in nine studies, of which six stated that it was given to adults with heart failure. According to Medscape (WebMD, NY, USA), heart failure is a ‘black box warning’ for thiazolidinediones because they can cause or exacerbate congestive heart failure in some patients. That is why these medications are not recommended for a patient with symptomatic heart failure and their initiation in a patient with established NYHA class III or IV heart failure is contraindicated.[44].

Metabolism and clearance of many of the hypoglycaemic agents is via the kidneys [45]. As a result, prescribing of antidiabetic medications for people with renal impairment is challenging. One of the hypoglycaemic agents that needs special emphasis in the presence of renal impairment is metformin. It is excreted unchanged through the kidneys and accumulates if renal function is impaired. Previously, metformin was contraindicated in males with serum creatinine > 1.5 mg/dL and females with serum creatinine > 1.4 mg/dL. In 2016 the US Food and Drug Administration (FDA) indicated that metformin should be avoided if estimated glomerular filtration rate (eGFR) is < 30 mL/min/1.73m^2^ [46]. The American Association of Clinical Endocrinologists/American College of Endocrinology (AACE/ACE), National Institute for Health and Care Excellence (NICE) and Australian Diabetes guidelines recommend stopping metformin if GFR < 30 mL/min/1.73m^2^ [47–49]. Mixed results were reported from the included studies regarding the GFR limit used to consider metformin use as inappropriate in an adult with decreased renal function. Huang et al. considered GFR < 60 [50], Lamine et al. and Diab took < 45 [51], (Diab MI, unpublished), and other studies considered a GFR of < 30 mL/min/1.73m^2^ [52, 53] as the contraindicated level of kidney function for metformin. This difference arose because authors used different criteria as a reference to decide on metformin contraindications.

Beta-blocker use in a diabetic patient with frequent hypoglycaemic episodes is not recommended because of the high risk of β-blockers causing hypoglycaemia, masking hypoglycaemic symptoms and decreasing hypoglycaemic awareness [54]. Six of the reviewed studies reported that the use of β-blockers in those with diabetes mellitus and frequent hypoglycaemic episodes is PIP [25, 26, 28, 31, 34, 43].

### Management of hypertension for adults with DM

Treatment of hypertension to a blood pressure target of < 140/90 mmHg reduces microvascular and cardiovascular events in people with DM [55]. Some of the included studies [14, 56], (Soorapan S, unpublished) reported that adults with diabetes and hypertension were not prescribed antihypertensive therapy. The American Diabetes Association (ADA), 2019 guideline recommends that diabetic patients with confirmed office-based BP ≥ 140/90 should be initiated with antihypertensive treatment. Moreover, ADA recommends starting two antihypertensive agents for people with stage II (≥ 160/100) hypertension to control BP more effectively. In line with this, Ayele et al. reported that monotherapy for people with a stage II hypertension was inappropriate [14]. Initiating two antihypertensive agents for adults with stage I hypertension is also unnecessary because a single agent is usually sufficient to control blood pressure [14]. Similarly, the ADA 2019 guideline recommends starting a single agent for people with an initial blood pressure record of < 160/100 mmHg [22].

The omission of ACEIs/ARBs in adults with diabetes and nephropathy or albuminuria was considered PIP in many of the included studies [24–26, 30–34, 57, 58], (Diab MI, unpublished; Aketchi IE, unpublished). Diabetic nephropathy occurs in 20–25% of people with T2DM and is the leading cause of end stage renal disease (ESRD) [59]. ACEIs and ARBs are the preferred agents for treatment of high blood pressure in people with diabetes and urine albumin to creatinine ratio (UACR) ≥ 30 or eGFR < 60 mL/min/1.73 m^2^, as these protect against kidney disease progression [49].

### Prevention and management of atherosclerotic cardiovascular disease (ASCVD)

People with diabetes have a higher risk of ASCVD as compared to non-diabetic individuals [60]. Lipid lowering therapy is recommended in people with diabetes and a prior CVD or high risk for CVD [22, 23]. A meta-analysis supports the use of a high- or moderate intensity statin as a first line therapy for LDL cholesterol lowering and cardio-protection [61]. Statins have been demonstrated to reduce the risk of CV events and death in people with DM when given as primary or secondary prevention [61, 62]. Consequently, not prescribing a statin therapy to an eligible adult with DM was considered as inappropriate in 28 included studies [2, 24–34, 58, 63–74], (Diab MI, unpublished; Aketchi IE, unpublished; Langenhoven W, unpublished).

People with DM and an established ASCVD should be given an antiplatelet agent as a secondary prevention strategy unless there is a clear contraindication. Most of the diabetes guidelines recommend low dose aspirin (75–350 mg) for this group of people [22, 23, 49]. Omission of antiplatelet therapy (e.g. aspirin) for adults with diabetes and CVD is reported by some of the studies in this review [29, 73, 75–79], (Soorapan S, unpublished; Aketchi IE, unpublished). Use of aspirin as a primary prevention may lead to more benefit than harm in people with high risk of CVD. The ADA guideline recommends the use of aspirin as a primary prevention for people with diabetes and age ≥ 50 with at least one additional major risk factor (family history of premature ASCVD, hypertension, dyslipidaemia, smoking, or chronic kidney disease/albuminuria). Similarly, many of the reviewed studies considered omission of antiplatelet therapy (e.g. aspirin) in adults with DM who are at risk of cardiovascular disease as PIP [24–27, 29–34, 56–58, 63, 75–85], (Soorapan S, unpublished; Langenhoven W, unpublished).

Inappropriate prescribing, especially in adults with diabetes, can result in increased morbidity and mortality and increased burden on the healthcare system and costs. Therefore, strategies to prevent and resolve PIP should be sought. Using an explicit tool to identify PIP which can be used as a physicians’ desk reference is a less costly and easy to use strategy to prevent PIP. Tools exist to assess PIP in older people and new tools and criteria frequently emerge. However, they do not specifically target people with diabetes. Only a few items relating to diabetes are usually included in these criteria. We strongly recommend the development of an up-to-date explicit tool that can be used to specifically address PIP for adults with diabetes.

This review included a large number of studies conducted on PIP among people with DM without restrictions to the study area, year and language of publication. Not conducting a critical appraisal of included studies is a limitation of this review and may have resulted in the inclusion of low-quality studies.

## Conclusion

PIP is common among people with DM. Contraindications, prescribing omissions and dosing problems were the most commonly reported PIPs. The specific events and conditions that were considered as inappropriate were inconsistent across included studies. PIP was less studied in low- and middle-income countries. There are no explicit criteria specifically designed to measure PIP for adults living with DM. Future studies focusing on the development of explicit tools to identify PIP for adults with DM are needed.

## Electronic supplementary material

Below is the link to the electronic supplementary material.

Supplementary Material 1
