# Supplementary material for: Potentially inappropriate prescribing for adults living with diabetes mellitus: a scoping review
Source: Int J Clin Pharm. 2022 Jul 1;44(4):860–72. doi: 10.1007/s11096-022-01414-7 (PMC9393152; doi:10.1007/s11096-022-01414-7)
Supplement: Supplementary file 1 — Supplementary Material 1 [file 11096_2022_1414_MOESM1_ESM.pdf]

Electronic Supplementary Material

Potentially inappropriate prescribing for adults living with diabetes mellitus: A scoping review

International Journal of Clinical Pharmacy

Mohammed Biset Ayalew[1, 2], Joy M. Spark[1], Frances Quirk[3], Gudrun Dieberg[4]

1. Pharmacy, School of Rural Medicine, University of New England, Armidale 2351, Australia
2. Department of Clinical Pharmacy, School of Pharmacy, University of Gondar, Gondar, Ethiopia
3. Faculty of Medicine and Health, University of New England, Armidale 2351, Australia
4. Biomedical Science, School of Science and Technology, University of New England, Armidale 2351, Australia

Corresponding author

Associate Professor Gudrun Dieberg
Biomedical Science
School of Science and Technology
University of New England
NSW 2351
Australia
Email – gdieberg@une.edu.au

Table of Contents

Supplementary Table 1: Detailed search strategy for PubMed database 2

Supplementary Table 2: Characteristics (extracted data) included studies 3

Supplementary Table 3: Specific PIP events reported in included studies 30

References 40

Supplementary Table 1: Detailed search strategy for PubMed database

	Search terms
#1	("Inappropriate Prescribing"[Mesh] OR "Medication Errors"[Mesh] OR "Contraindications, Drug"[Mesh])
#2	Title/Abstract search – key words for inappropriate prescribing (inappropriate prescri*[Title/Abstract] OR medication error*[Title/Abstract] OR inappropriate medic* use[Title/Abstract] OR prescri* omission*[Title/Abstract] OR drug related problem*[Title/Abstract] OR drug therapy problem*[Title/Abstract] OR appropriat* prescri*[Title/Abstract] OR overprescri*[Title/Abstract] OR underprescri*[Title/Abstract] OR misprescri*[Title/Abstract] OR drug interact*[Title/Abstract] OR dos* error*[Title/Abstract] OR contraindicat*[Title/Abstract] OR unnecessary drug therap*[Title/Abstract] OR ineffective drug*[Title/Abstract] OR medication use[Title/Abstract] OR omission[Title/Abstract])
#3	("Diabetes Mellitus, Type 1"[Mesh] OR "Diabetes Mellitus, Type 2"[Mesh] OR "Hyperglycemia"[Mesh] OR "Hypoglycemic Agents"[Mesh])
#4	(diabet*[Title/Abstract] OR hyperglycemi*[Title/Abstract] OR hypoglycemic agent*[Title/Abstract] OR antidiabet*[Title/Abstract] OR high blood glucose [Title/Abstract])
#5	#1 OR #2
#6	#3OR #4
#7	#5 AND #6

Supplementary Table 2: Characteristics (extracted data) included studies

Author Year Country	Article type	Study design	Study population	Study setting	Total sample size	DM cases	Criteria for PIP	Type of PIP	Prevalence of PIP (in %) [a]	Involved medications
Abdulmalik 2019 Ethiopia[1]	Original article	RCS	Adults with T2DM	OP	148	148	Ethiopian Treatment Guideline	DP IDS PO UDT	DP = 37.8 IDS = 19.7 PO = 13.4 UDT = 19.7 of DRPs	metformin, glibenclamide, insulin
Abu Farha 2019 Jordan[2]	Original article	RCS	Adults with T2DM	OP	91	91	Lexicomp, I-fact software	CI PO UDT	NR	NR
Ahmad 2014 Netherlands[3]	Original article	PCS	Older adults (> 60 years) using ≥ five prescription drugs	IP	340	73	Tool developed by Mast et al (unpublished data, 2013)	CI DP PO	NR	glibenclamide, metformin, ACEIs, statins, pioglitazone, β-blockers, thiazides
Ahmed 2020 Ethiopia[4]	Original article	CS	Adults with DM	PO	100	100	NR	DP	DP = 63	NR
Aketchi 2014 Kenya[5]	Thesis	RCS	Adults with T2DM	OP	369	369	PQI (Martirosyan et al, 2008), and Beers Criteria (2012)	CI DDI PO	DDI = 4	long-acting sulfonylurea, aspirin, antihypertensive drugs, β-blockers, statins, ACEIs/ARBs, metformin, thiazide
Al Aqqad 2014 Malaysia[6]	Original article	PC	Older adults (≥65 years)	NH	211	57	STOPP Criteria	CI	NR	glibenclamide, chlorpropamide, β-blockers
Al Khaja 2018 Bahrain[7]	Original article	CS	Older adults with HTN ± DM, prescriptions	OP	2090	1073	STOPP Criteria (version 1)	CI IDS UDT	CI = 2.3 IDS = 0.74 UDT = 3.82 of prescriptions	glyburide, furosemide, dipyridamole, diuretics, RAAS inhibitors

Alauddin 2020 USA[8]	Original article	RC	Adults with T2DM	IP	200	200	SMPC	CI	CI = 1.0	metformin
Ali 2015 Pakistan[9]	Case report	CS	Adults with DM	IP	15	15	Micromedex, Drugs.com	DDI<br>DP<br>PO<br>UDT	DDI = 60.06<br>DP = 9.1<br>PO = 6.1<br>UDT = 6.1	insulin, diuretics, β-blockers
AL-Musawe 2020 Portugal[10]	Original article	PCS	Older adults with T2DM	NR	670	670	Micromedex, STOPP (version 2)	CI<br>DDI	CI = 36.1<br>major DDI = 10.6	ACEIs/ARBs, aspirin, clopidogrel, CCBs, simvastatin, HCT, pravastatin, lisinopril
Al-Taani 2017 Jordan[11]	Original article	PCS	Adults with DM	OP	1494	1494	MAI, national and international guidelines and compendium, Lexicomp Drug Information Handbook	DP<br>IDS<br>PO<br>UDT	DP = 10.6<br>IDS = 16.7<br>PO = 39.2<br>UDT = 26.1	NR
Alyazeedi 2019 Qatar[12]	Original article	RC	Older adults	OP	3537	2129	Beers Criteria (2019)	CI	CI = 70.8	glibenclamide, insulin aspart, lispro and regular insulin
Anderson S 2020 USA[13]	Original article	PC	Adults with DM, not on statin therapy	OP	326	326	ACC/AHA cholesterol guidelines	PO	PO = 84.8	statins
Anderson T 2020 USA[14]	Original article	RC	Older adults with DM	IP	1626	1626	NR	DP<br>UDT	PIP = 49 of patients receiving intensification	NR

Andreassen 2016 UK[15]	Original article	RCS	Adults with T2DM	NH	826	106	Medicines optimisation tool "Optimising Safe and Appropriate Medicines Use"	IDS	IDS = 90.6	NR
Arafath 2015 India[16]	Original article	RCS	Adults with DM	IP	148	148	NR	DDI	DDI = 46.6	glipizide, aspirin, enalapril, insulin, metformin, nifedipine, pioglitazone
Araújo 2013 Brazil[17]	Original article	PCS	Adults with T2DM	OP	579	579	NR	DDI	NR	antidiabetics (metformin, glibenclamide, arcabose), diuretics, ACEIs, anti-lipidemic drugs
Araújo 2019 Brazil[18]	Original article	PCS	Adults with DM and/or HTN	CD	558	266	Beers Criteria (2015)	CI	CI = 22.9	NR
Ayele 2018 Ethiopia[19]	Original article	RCS	Adults with T2DM and HTN	OP	203	203	Drug Information Handbook, Standards of Medical Care in Diabetes (2017) and JNC 8	PO UDT	PO = 40.7 UDT = 10.7 of DRPs	NR
Babu 2016 India[20]	Original article	PCS	Adults with DM, with comorbidities/ complications	IP	102	102	Micromedex	DDI	NR	insulin, metformin, aspirin
Basheti 2017 UAE[21]	Original article	PCS	Adults with chronic diseases	OP	125	30	Lexicomp Drug Information Handbook (22nd ed)	DDI DP IDS PO	DDI = 6.7 DP = 16.7 IDS = 13.3 PO = 13.3	NR
Belaiche 2012 France[22]	Original article	PCS	Adults with CKD	OP	67	51	Act-IP document - French Society of Clinical Pharmacy	DP	DP = 9.8	NR

Bezabhe 2020 Australia[23]	Original article	RCS	Adults with CKD	OP	44259	15263	PQIs (Smits et al.) [b]	CI	CI = 14.1 of patients with eGFR<30mL	metformin
Blundell 2015 Spain[24]	Original article	Interv	Older adults (≥ 65 years), poly-medicated	OP	84	NR	STOPP/START, CheckTheMeds software	CI PO	NR	glyburide, chlorpropamide, β-blockers, metformin, ACEIs/ARBs, antiplatelet agents, statins
Borges 2010 Brazil[25]	Original article	Interv	Adults with T2DM	OP	64 PC group = 33 Control = 31	64	Pharmacist's Workup of Drug Therapy (University of Minnesota),	DP IDS PO	DP = 33.3 IDS = 9.1 PO = 33.3 of PC group	NR
Breuker 2017 France[26]	Original article	PCS	Adults	IP	904	671	NR	DP PO UDT	DP = 12.8 PO = 19.7 UDT = 1.3 at admission	hypoglycaemic sulfamide, insulin, glargine
Bulatova 2007 Jordan[27]	Original article	PCS	Adults with DM, eligible for antiplatelet prophylaxis	OP	199	199	ADA guideline (2003)	PO	PO = 16	antiplatelet agents (aspirin, clopidogrel or ticlopidine)
Calabrese 2002 USA[28]	Original article	RCS	Adults on at least 1 dose of metformin	IP	204	204	SMPC of metformin	CI	CI = 31	metformin
Capafons 2005 Spain[29]	Original article	PC	Adults with DM, obese, on metformin	IP	135	135	SMPC of metformin	CI	CI = 14.8	metformin
Casparie 1985 Netherlands[30]	Original article	PC	Adults with DM and severe hypoglycaemic events	IP/OP	26	26	Algorithms for adjustment of insulin dosage (Skyler, et al., 1981)	DP IDS	NR	insulin

Castro-Ríos 2008 Mexico[31]	Original article	Interv	Adults with T2DM, on hypoglycaemics	OP	625	625	Clinical practice guidelines	DP IDS	NR	glibenclamide, metformin
Caughey 2010 Australia[32]	Original article	RCS	Veterans (≥ 65 years), on at least one ADM	IP/OP	18968	18968	Beers Criteria (2003), Australian Evidence-Based Guidelines for the Management of T2DM (2005)	CI	CI = 22.7	metformin, glibenclamide, glimepiride, TZD, β-blockers, drugs in Beers' list
Caughey 2016 Australia[33]	Original article	RC	Older adults with DM	IP	876	876	Beers Criteria (2012)	CI	CI = 49.4 before - CI = 45.5 after - hospitalisation	sulphonylureas (glibenclamide or glimepiride), α1-blockers
Chin 2015 Pakistan[34]	Original article	RCS	Adults with DM	IP	235	235	Micromedex	DDI	DDI = 39.1	insulin, duloxetine, bisoprolol, aspirin, lisinopril, metformin
Chou 2013 Taiwan[35]	Original article	RCS	Prescriptions with special oral formulations	OP	124,300	14428	SMPC	IDS	IDS = 3.1	gliclazide, glipizide, metformin
Christiaens 2020 Belgium[36]	Original article	RC	Older adults with T2DM	IP	318	318	Endocrine Society guideline 2019	DP PO UDT	57.2% over-, 17.9% under-treatment	NR
Chung 2017 Hong Kong[37]	Original article	PCS	Older adults with DM (≥ 65 years), high risk (multiple disease state & complex drug regimen)	OP	522	522	NR	CI DDI DP IDS PO UDT	CI = 1.2 DDI = 1.0 DP = 43.9 IDS = 2.4 PO = 8.4 UDT = 5.3 of DRPs	NR
Courtenay 2007 UK[38]	Original article	Interv	Adults with DM treated with insulin ± OHA	IP	452	452	NR	DP PO	NR	insulin, OHA
De Arau´jo 2020 Brazil[39]	Original article	PC	Older adults	CD	418	98	Beers Criteria 2019	CI	PIP = 45.9	glibenclamide, glimepiride

Demoz 2019 Ethiopia[40]	Original article	PCS	Adults with DM	OP	418	418	NR	CI DP IDS PO UDT	CI = 1.9 DP = 30.4 IDS = 26.1 PO = 25.1 UDT = 9.7 of DRPs	NR
Devalia 2010 UK[41]	Original article	RCS	Adults with DKA	IP	36	36	Trust DKA protocol	DP IDS	NR	insulin
Devetzis 2011 Greece[42]	Case report	Crosssectional	Adults with T2DM and MALA	IP	2	2	NR	CI	NR	metformin
Diab 2012 Qatar[43]	Thesis	RCS	Adults with T2DM and no history of CVD	OP	305	305	MAT	CI DDI IDS PO UDT	NR	statins, metformin, sulfonylureas, DPP-4 inhibitors, pioglitazone, meglitinides, TZD, ACEIs/ARBs, insulin
Dias 2019 Brazil[44]	Original article	PCS	Older adults	IP	255	110	Micromedex	DDI	DDI = 81.8	NR
Dinesh 2007 Nepal[45]	Original article	PCS	Adults with DM	OP	182	182	Micromedex	DDI	DDI = 52.2	metformin, enalapril, atenolol, aspirin, amlodipine, gliclazide, glibenclamide, insulin
Doellner 2017 USA[46]	Original article	RC	Older adults and Medicare beneficiaries (≥ 65 years)	CD	200	126	NR	CI PO	NR	statins, sulfonylurea
Dongre 2019 India[47]	Original article	PCS	Adults with T2DM	IP	106	106	Micromedex, Medscape and drugs.com	DDI	DDI = 59.4	metformin, furosemide, spironolactone, glimepiride, metoprolol

Dosa<br>2013<br>USA[48]	Original article	RCS	Older adults, veterans<br>(≥ 65 years)	NH	27133	10776	Health Plan Employer Data and Information Set (HEDIS)	CI	CI = 10.2	NR
Ellis<br>2015<br>USA[49]	Original article	RC	Adults with painful DPN, on pregabalin (P) or duloxetine (D)	IP/OP	892<br>P = 446<br>D = 446	892	Micromedex	DDI	DDI = 56.7 for duloxetine subjects	pregabalin, duloxetine
Elnaem<br>2017<br>Malaysia[50]	Original article	RCS	Adults with T2DM<br>(40-75 years)	IP	393	393	Malaysian Clinical Practice Guidelines	DDI<br>DP<br>PO	DDI = 26<br>DP = 5.6<br>PO = 35.1	statins, amlodipine, colchicine, verapamil, warfarin
Elnaem<br>2019 (a)<br>Malaysia[51]	Original article	RCS	Adults with T2DM eligible for lipid-lowering therapy	OP	816	816	Malaysian Clinical Practice Guidelines	DDI<br>DP<br>IDS<br>PO	DDI = 17.5<br>PO = 12.4<br>of prescriptions	amlodipine, gemfibrozil, diltiazem, verapamil, digoxin, amitriptyline, statins
Elnaem<br>2019 (b)<br>Malaysia[52]	Original article	Interv	Adults with T2DM	IP/OP	1598	1598	Malaysian Clinical Practice Guidelines for T2DM	DDI<br>DP<br>IDS<br>PO	PIP = 45.6	statins
Emslie-Smith<br>2001<br>UK[53]	Original article	RC	Adults with T2DM	IP	1847	1847	BNF, SMPC	CI	CI = 24.5	metformin
Erah<br>2013<br>Nigeria[54]	Original article	PCS	Prescribers for older adults with DM	OP	149	NR	Beers Criteria (2012)	CI<br>DP	NR	chlorpropamide, glibenclamide, glyburide
Faragon<br>2003<br>USA[55]	Original article	Interv	Adults with DM	OP	85	85	ADA guideline	PO	PO = 55.3	aspirin
Formiga<br>2016<br>Spain[56]	Original article	PCS	Older adults<br>(75 years)	IP	672	249	Beers Criteria, STOPP/START Criteria, ACOVE-3 tool	CI<br>DP<br>PO	NR	digoxin, amiodarone, CCBs, ACEIs, statins

Frankenthal 2015 Israel[57]	Original article	RCS	Older adults (≥65 years)	IP	300	96	STOPP/START Criteria	CI PO	NR	glibenclamide, metformin, β-blockers, ACEIs/ARBs, antiplatelet agents, statins
Fu 2011 USA[58]	Original article	RC	Adults with T2DM (≥25 years old) or prescribed with ADM	OP	125464	125464	ADA guideline (2008)	PO	PO = 35.7	statins
Gagnon 2020 Canada[59]	Original article	RC	Older adults with DM	NR	286962	286962	Beers criteria (2015)	CI DP	PIP = 56.1	glibenclamide
Galván-Banqueri 2013 Spain[60]	Original article	PCS	Adults with multiple comorbidities	NR	244	NR	STOPP/START Criteria, MAI	DDI PO	NR	statins, antiplatelet agents, metformin, glyburide, β-blockers, chlorpropamide
Garat 2010 France[61]	Case report	RCS	Adults with T2DM and metformin serum concentration > 2.3 mg/L	NR	30	30	NR	CI	CI = 93.4 of cases	metformin
Giorda 2020 Italy[62]	Original article	RCS	Adults with T2DM	OP	510	510	Italian Standards for Treatment of DM 2016	CI	CI = 70.6	sulfonylureas
Gor 2019 USA[63]	Original article	RCS	Adults with T2DM	NR	3237	3237	NKF KDOQI guideline	CI	CI = 4.9 for patients onmetformin (eGFR<30) CI = 18.4 on metformin (SCr>1.4/1.5) CI = 12.3 on glyburide (eGFR<60)	metformin, glyburide

Granas<br>2010<br>Norway[64]	Original article	RCS	Adults with T2DM	OP	73	73	NR	DDI<br>DP<br>IDS<br>PO<br>UDT	NR	antidiabetics, lipid-lowering agents, β-blockers, diuretics, ACEIs/ARBs, antithrombotic agents
Haggerty<br>2005<br>USA[65]	Original article	Interv	Adults with DM	OP	322	322	ADA guideline (2002)	PO	PO = 24.5	aspirin
Harder<br>2009<br>Germany[66]	Original article	CS	Adults with more than 3 chronic prescriptions & at least 1 ADM	OP	102	102	SMPC	CI<br>DDI<br>DP<br>PO	CI = 15<br>DDI = 10<br>DP = 3	β-blockers, ACEIs/ARBs, antiplatelet agents, statins
Hartuti<br>2019<br>Indonesia[67]	Original article	PCS	Adults with T2DM	IP	81	81	Medscape, Micromedex, guidelines (ADA, AACE, NICE)	DDI<br>DP<br>IDS<br>PO	DDI = 45.6<br>DP = 32.4<br>IDS = 4.4<br>PO = 2.9	metformin, insulin
Haugbølle<br>2006<br>Denmark[68]	Original article	PCS	People with T2DM, angina pectoris and asthma	OP	414	192	NR	DDI<br>DP<br>IDS	DDI = 0.6<br>DP = 8.2<br>IDS = 9.1<br>of DRPs	insulin, metformin
Herman<br>2016<br>Indonesia[69]	Original article	RCS	Older adults with T2DM	IP	50	50	STOPP/START Criteria	CI	CI = 30	glibenclamide
Hinds<br>2016<br>USA[70]	Original article	RCS	Adults with DM (40-75 years)	OP	583	583	Blood Cholesterol Guideline (2013)	DP	DP = 41.3	statins
Holstein<br>1999<br>Germany[71]	Original article	CS	Adults with T2DM on metformin	IP	308	308	SMPC, Handbook of Experimental Pharmacology (Vol 119), ADA guideline (1996)	CI	CI = 73	metformin

Hong<br>2020<br>South Korea[72]	Original article	RC	Adults with T2DM and CKD	NR	82,332	82,332	SMPC of metformin	DP	DP = 26.2	DPP-4 inhibitors
Huang D<br>2014<br>USA[73]	Original article	RCS	Older adults (≥65 years) on metformin	OP	356	356	SMPC (2013), FDA, American Geriatrics Society (AGS) guideline	CI	CI = 31.4	metformin
Huang W<br>2014<br>Australia[74]	Original article	RCS	Adults with T2DM on metformin	IP	301	301	AMH (2013), Diabetes Australia: Current Guidelines for the Management of T2DM	CI<br>DP<br>IDS	CI = 21.6<br>DP = 16.5<br>IDS = 31	metformin
Huang W<br>2015 (a)<br>Australia[75]	Original article	RCS	Lactic acidosis cases associated with metformin	OP	152	132	NR	CI	CI = 26.5 of cases	metformin
Huang W<br>2015 (b)<br>Australia[76]	Original article	RCS	Adults with T2DM on metformin	CD	6386	6386	AMH (2014)	CI<br>DP	CI = 8.6<br>DP = 6.7	metformin
Huri & Ling<br>2013<br>Malaysia[77]	Original article	RCS	Adults with T2DM and dyslipidaemia	IP/OP	208	208	Beers Criteria, Malaysian Clinical Practice Guidelines	CI<br>DDI<br>DP<br>IDS<br>PO<br>UDT	CI = 3.7<br>DDI = 18<br>DP = 14.3<br>IDS = 6.9<br>PO = 11.3<br>UDT = 4.2<br>of DRPs	lipid-lowering agents, antidiabetics, antihypertensives, antiplatelet agents, cardiovascular agents
Huri & Wee<br>2013<br>Malaysia[78]	Original article	RCS	Adults with T2DM and HTN	IP	200	200	ADA guideline (2012), JNC 7, Malaysian Clinical Practice Guidelines	CI<br>DDI<br>DP<br>IDS<br>PO<br>UDT	CI = 7.5<br>DDI = 16.3<br>DP = 16<br>IDS = 9.8<br>PO = 3.9<br>UDT = 1.3<br>of DRPs	aspirin, clopidogrel, simvastatin, amlodipine, metformin

Ibrahim<br>2005<br>USA[79]	Original article	RCS	Adults with DM receiving home health care services	CD	139	139	Micromedex	DDI	major DDI<br>= 38.8<br>moderate DDI<br>= 92.8<br>mild DDI<br>= 70.5	diuretics, antihypertensives, cardiac agents
Ikaheimo<br>2019<br>Finland[80]	Original article	PCS	Older adults with/out T2DM (≥ 65 years)	CD	358	182	SFINX-PHARAO database	DDI	DDI = 44.5	ACEIs/ARBs, platelet aggregation inhibitors, β-blockers, diuretics, statins
Izquierdo<br>2007<br>USA[81]	Original article	Interv	Older adults with DM<br>(≥ 55 years)	CD	338	338	Physician Desk Reference, VHA Clinical Practice Guidelines (2000) and ADA guideline (2005)	CI<br>DP	CI = 7.1<br>DP = 2.4	metformin, TZD
Jameson<br>2016<br>UK[82]	Original article	RC	Adults with T2DM, prescribed GLP-1 receptor agonist	OP	7133	7133	NICE guidelines	IDS	IDS = 75	GLP-1 receptor agonist (liraglutide, exenatide)
Jiang<br>2003<br>Georgia[83]	Thesis	RCS	Older adults<br>(≥ 65 years)	NH	1161	310	Beers Criteria (1997)	IDS	IDS = 42.9	
Johnston<br>2013<br>USA[84]	Original article	RC	Adults with DPN on either pregabalin (P) or duloxetine (D)	OP	P = 2499<br>D = 1354	P = 2499<br>D = 1354	Micromedex	CI<br>DDI	NR	pregabalin, duloxetine
Kara<br>2016<br>Turkey[85]	Original article	PCS	Older adults<br>(≥ 65 years)	IP/OP	374	160	STOPP/START Criteria	CI<br>PO	NR	β-blockers, metformin, ACEIs, antiplatelet agents, statins
Karandikar<br>2013<br>India[86]	Original article	PCS	Older adults<br>(≥ 65 years)	IP/OP	600	NR	STOPP/START Criteria and Beers criteria	CI<br>PO	CI = 5.9<br>PO = 13.4<br>of DRPs	statins, metformin, β-blockers

Kassam<br>2007<br>Canada[87]	Original article	RCS	Older adults with DM<br>(≥ 70 years)	OP	138	138	Canadian Diabetes Association (2003) clinical practice guidelines	DDI<br>DP<br>PO<br>UDT	DDI = 0.4<br>DP = 12.7<br>PO = 49.3<br>UDT = 2.5<br>of DRPs	NR
Kavousi<br>2019<br>India[88]	Original article	PCS	Adults with DM	IP	110	110	Guidelines	DP	DP = 6.36	NR
Kefale<br>2020<br>Ethiopia[89]	Original article	CS	Adults with T2DM and HTN	OP	423	423	NR	CI<br>DP<br>IDS<br>PO<br>UDT	CI = 0.8<br>DP = 19.9<br>IDS = 4.3<br>PO = 24.2<br>UDT = 8.4 of DRPs	metformin, glibenclamide, β-blockers, statins, aspirin
Khalil<br>2018<br>Australia[90]	Original article	RCS	Adults with T2DM<br>on ADM	IP	206	206	Prescribing guidelines in MIMS	CI	NR	metformin, insulin, sulphonylureas, DPP-4 inhibitors
Khamaisi<br>2012<br>Israel[91]	Case report		A 70-year-old patient with well-controlled T2DM	OP	1	1	NR	DDI	NR	repaglinide and brotizolam
Klinke<br>2004<br>Canada[92]	Original article	PCS	Adults with T2DM living in rural regions	CD	342	342	ADA guideline (2003), Canadian Diabetes Association (CDA) clinical practice guidelines (1998)	PO	NR	aspirin
Kochetkov<br>2019<br>Russia[93]	Original article	RCS	Older adults with T2DM & HTN (≥ 65 years)	IP	260	260	STOPP/START Criteria	CI<br>PO	CI = 22.3<br>PO = 68.5	sulfonylureas, loop diuretics, β-blockers, statins, clopidogrel, ACEIs/ARBs, aspirin
Kolawole<br>2004<br>Nigeria[94]	Original article	RCS	Adults with T2DM	OP	82	82	ADA guideline (2003)	CI<br>PO	CI = 1.2<br>PO = 59.8	aspirin

Korhonen<br>1979<br>Finland[95]	Original article	RCS	Older adults (≥ 65 years) with biguanide induced lactic acidosis	IP	24	24	NR	CI	NR	phenformin, metformin
Kosmalski<br>2012<br>Poland[96]	Original article	PCS	Adults with poorly controlled T2DM	IP	558	558	NR	CI	CI = 49.3	metformin
Kovacevic´<br>2014<br>Serbia[97]	Original article	PCS	Older adults (≥ 65 years), collected prescribed medication at a community pharmacy	OP	509	148	STOPP/START Criteria	CI<br>PO	CI = 4.1<br>PO = 91.2	glibenclamide, metformin, ACEIs, antiplatelet, statins
Krishnarajan<br>2020<br>India[98]	Original article	PC	Adults with T2DM	OP	203	203	NR	DDI	DDI = 18.7	glimepiride, pioglitazone, insulin, glargine
Kumar<br>2011<br>India[99]	Original article	PCS	Adults with T2DM	IP	142	142	Text books and the drug interaction facts software V4.0	DDI	NR	atorvastatin, ACEIs, clopidogrel, digoxin, aspirin, enoxaparin, furosemide, insulin, thiazide, timolol
Kumar<br>2018<br>India[100]	Original article	PCS	Adults with T2DM and HTN	IP	100	100	Updated evidence-based disease management guidelines	DDI<br>DP<br>IDS<br>PO<br>UDT	DDI = 18.2<br>DP = 4<br>IDS = 14.2<br>PO = 2<br>UDT = 4.5<br>of DRPs	NR
Kwong<br>1998<br>USA[101]	Case report	CS	67-year-old man with DM	IP	1	1	NR	CI	NR	phenformin

Lahoz 2007 Spain[102]	Original article	PCS	Adults (18-70 years) receiving statin treatment	OP	1817	594	NCEP-ATP III guideline and the European Guidelines on CVD Disease Prevention	UDT	UDT = 1	statins
LaMarr 2010 USA[103]	Original article	Interv	Adults with DM, eligible for ACEIs/ARBs or aspirin	OP	71 (asprin) 70 (ACEIs/ ARBs)	71 (asprin) 70 (ACEIs/ ARBs)	ADA guideline (2009)	PO	NR	ACEIs/ARBs, aspirin
Lamine 2016 Switzerland[104]	Original article	CS	Adults with T2DM	OP	1359	1359	SSED clinical practice guidelines	CI	NR	metformin, sulfonylurea, GLP-1 agonist, DPP-4 inhibitor
Landi 2007 Italy[105]	Original article	PC	Older adults (≥ 80 years)	CD	364	109	Beers criteria (2003)	NR	PIP = 33	NR
Langenhoven 2015 South Africa[106]	Thesis	RCS	Adults with T2DM	OP	300	300	SEMDSA guidelines	PO	NR	ACEIs, aspirin, statins
Lati 2020 Kenya[107]	Original article	PCS	Adults with DM and HTN	OP	104	104	Micromedex	DDI	NR	β-blockers, enalapril, metformin
Laurent 2019 France[108]	Original article	PCS	Adults with T2DM	IP	228	228	Pharma software	DDI	DDI = 61.8	insulin, β-blockers, glinides, gliptins, sulfamides
Laville 2018 France[109]	Original article	PC	Adults with CKD (stages 3 to 5)	OP	3033	1295	SMPC, European Renal Best Practice	CI DP	CI = 7.6 DP = 13.7 of patients on antidiabetic drugs (n=1085)	NR
Leitao 2006 Brazil[110]	Original article	PCS	Adults with T2DM (> 40 years)	OP	636	636	ADA guideline	PO	PO = 72.5	aspirin

Leonard<br>2016<br>USA[111]	Original article	RC	Adults with DM on sulfonylureas & statins/fibrates	OP	592872	592872	NR	DDI	NR	glyburide, glipizide, glimepiride, statins, fenofibrate, gemfibrozil
Lian<br>2008<br>UK[112]	Thesis	RCS/<br>PCS	Adults with DM	OP	47	47	MAT	DP<br>IDS<br>PO<br>UDT	DP = 38.5<br>IDS = 1.4<br>PO = 21.7<br>UDT = 0.7<br>of DRPs	NR
Lindblad<br>2005<br>USA[113]	Original article	CS	Frail older adults (≥ 65 years)	IP	397	NR	Combined consensus explicit criteria from Beers and McLeod	CI	NR	β-blockers
Liu<br>2012<br>Taiwan[114]	Original article	RCS	Older adults (≥ 65 years) discharged from medical wards	IP	520	194	STOPP/START Criteria	PO	NR	metformin, antiplatelet agents, statins
Lockery<br>2020<br>Australia/USA[115]	Original article	NR	Older adults	CD	19,114	2045	Beers Criteria 2019	CI	PIP = 45.2	long-acting sulfonylureas
Lopez<br>2014<br>Spain[116]	Original article	RCS	Older adults (≥ 65 years) attending clinic for ≥2 times	OP	247	NR	STOPP/START Criteria	CI<br>PO	NR	glyburide, metformin, ACEIs/ARBs, antiplatelet drugs, statins
Lu<br>2020<br>China[117]	Original article	RC	Adults with diabetic nephropathy	OP	1128	1128	ADA (2018) and Chinese guidelines	NR	PIP = 27.1 (ADA)<br>PIP = 31.9 (Chinese guideline)	NR
Luz<br>2018<br>Brazil[118]	Original article	CS	Adults (≥ 60 years)	IP	227	NR	Brazilian version of the START Criteria	PO	NR	metformin, ACEIs/ARBs, statins, antiplatelet agents
Maciulaitis<br>2006<br>Lithuania[119]	Original article	RCS	Adults with DM	IP/OP	906	906	National Guidelines on Prescription of Medicine	CI<br>IDS	NR	TZD, insulin

Maheshwari<br>2019<br>India[120]	Original article	RCS	Adults with T2DM	IP	138	138	SMPC, ADA guideline	CI	CI = 42	metformin
Mahner<br>2018<br>Germany[121]	Original article	PCS	Adults with CKD (stage ≥ 3)	OP	589	372	SMPC, recommendations of scientific societies and regulatory authorities	CI<br>DP	NR	metformin, sitagliptin, ACEIs, diuretics
Mahwi<br>2013<br>Iraq[122]	Original article	Interv	Adults with T2DM and HbA1c > 7.0%	OP	123	123	NR	DP<br>IDS<br>PO<br>UDT	DP = 58<br>IDS = 12.9<br>PO = 21<br>UDT = 21	NR
Maidana<br>2017<br>Paraguay[123]	Original article	Interv	Adults with T2DM	OP	61<br>I = 31<br>C = 30	61	NR	DDI<br>DP<br>PO	DDI = 11.3<br>DP = 27.6<br>PO = 23.8<br>of DRPs	NR
Manes<br>2006<br>Italy[124]	Original article	CS	Adults (> 35 years) in primary care center and on aspirin treatment	OP	400	93	Italian Cardiovascular Risk Chart for Primary Prevention, European Society of Cardiology Coronary Risk Chart	UDT	UDT = 3.2 (European Criteria)<br>UDT = 10.8 (Italian Criteria)	aspirin
Manley<br>2003<br>USA[125]	Original article	RCS	Ambulatory haemodialysis patients	OP	133	77	NR	DDI<br>DP<br>PO<br>UDT	DDI = 1<br>DP = 15.2<br>PO = 19.1<br>UDT = 29.7<br>of DRPs	NR
Margiani<br>2014<br>Italy[126]	Case report	CS	70-year-old Caucasian man with T2DM and HTN	IP	1	1	Italian Medicines Agency (AIFA) drug safety recommendation	CI	NR	metformin

Marroquin 2012 Spain[127]	Original article	PCS	Older adults (≥ 65 years)	OP	471	NR	STOPP/START Criteria	CI<br>PO	NR	glyburide, chlorpropamide, metformin, antiplatelet agents, aspirin, statins
Masoudi 2003 USA[128]	Original article	RCS	Adults with DM and HF	IP	25663	25663	SMPC of metformin and thiazolidinediones	CI	NR	metformin, TZD
Matsumura 2009 Japan[129]	Original article	Interv	Adults with liver/renal diseases and/or DM	IP	929	NR	SMPC, Renal Disease Resident Manual text book,	CI	NR	antidiabetics
McFarland 2009 USA[130]	Original article	RCS	Adults with DM who received initial sitagliptin dose	OP	290	290	SMPC	DP	DP = 12.1	sitagliptin
Mechessa 2020 Ethiopia[131]	Original article	CS	Adults with DM	OP	141	141	Micromedex, Medscape, ADA guideline	CI<br>DDI<br>DP<br>IDS<br>PO<br>UDT	CI = 3.8<br>DDI = 30.8<br>DP = 21.8<br>IDS = 7.7<br>PO = 6.4<br>UDT = 7.1	NR
Milligan 2011 UK[132]	Original article	RCS	Adults with DM	NH	768 error incidents		NR	DP<br>PO<br>UDT	DP = 11.7<br>PO = 16.5<br>UDT = 30.1	insulin, OHA
Mino-León 2018 Mexico[133]	Review	NR	Adults with T2DM and HTN, bone/joint diseases and depression	NR	NA	NA	NR	CI<br>DDI	NR	glibenclamide, sulfonylureas, duloxetine, aspirin, antiplatelet agents
Modesto 2020 Brazil[134]	Original article	CS	Severely obese adults	OP	150	60	Micromedex	DDI	DDI = 61.7	aspirin, metformin, enalapril, captopril

Molist-Brunet 2019 Spain[135]	Original article	PCS	Frail older adults with T2DM	IP	210	210	ADA guideline	CI DP IDS	PIP = 66.2	metformin, sulfonylureas, insulin, meglitinides
Mori 2017 Brazil[136]	Original article	CS	Older adults with CVD (≥ 60 years) on ≥ 1 medication prior to hospital admission	IP	230	113	STOPP/START criteria (version 1)	CI PO	CI = 16.8 PO = 49.6	glibenclamide or chlorpropamide, statins, antiplatelet agents, ACEIs/ARBs, metformin
Muller 2016 France[137]	Original article	PCS	Adults with T2DM and CKD	OP	301	301	Clinical practice guideline on DM and CKD	CI DP	NR	metformin, gliclazide, glibenclamide, glimepiride, repaglinide, acarbose, exenatide, liraglutide, vildagliptin, sitagliptin, saxagliptin
Mwita 2020 Botswana[138]	Original article	RCS	Adults with T2DM	OP	500	500	SEMDSA guidelines	PO	PO = 52	statins
Najim 2010 Malaysia[139]	Original article	PCS	Adults with T2DM, non-immuno-compromised on metformin	OP	1001	1001	Malaysian Clinical Practice Guidelines and SMPC	CI	CI = 33.6	metformin
Nelson 2018 UK[140]	Original article	RCS	Adults with DM and renal impairment, on OHA	OP	178	178	Renal licences of antidiabetic medications	CI DP	CI = 2.8 DP = 3.9	metformin, sitagliptin, gliclazide
Niehoff 2016 USA[141]	Original article	PCS	Older adults with DM & HTN (≥ 65 years) on ≥ 7 medications	OP	40	40	The Tool to Reduce Inappropriate Medications (TRIM), algorithms based on Beers	DP UDT	DP = 5 UDT = 43	NR

							Criteria (2012) and STOPP (2008)			
Nuñez-Montenegro 2019 Spain[142]	Original article	PCS	Older adults (≥ 65 years) on multiple medications	CD	425	224	STOPP/START Criteria	CI PO	CI = 48.2 PO = 54.5	NR
Ogamba 2016 Kenya[143]	Thesis	CS	Adults with T2DM and HTN	OP	168	168	Medscape drug interaction checker	DDI	DDI = 96	losartan, enalapril, HCT, pregabalin, atenolol, carvedilol, furosemide, captopril, nifedipine
Oktora 2020 Netherlands[144]	Original article	RCS	Adults with DM	OP	138814	138814	Beers criteria (2015), PROMPT	NR	PIP = 17.4	long-acting sulfonylureas
Pasina 2020 Italy[145]	Original article	RCS	Adults in long-term care	NH	2579	454	NR	PO UDT	PO = 26.7 UDT = 27.1	antiplatelet agents
Patel 2016 USA[146]	Original article	RCS	Adults with DM and HF, discharged from hospital	IP	8791	8791	NR	CI	NR	insulin, sulfonylureas, metformin, TZD, DPP4-inhibitors
Penfornis 2014 France[147]	Original article	PCS	Adults with T2DM for more than 1 year, treated with OHA and/or insulin	OP	3704	3704	National Kidney Foundation Clinical Practice Guideline for DM and CKD (2012), NICE clinical guideline (2009)	CI	NR	metformin, sulfonylurea, meglinides, DPP4-inhibitors
Pitkala 2002 Finland[148]	Original article	PCS	Older adults (≥ 75 years) urban residents	CD	2511	286	Beers Criteria (1997)	CI	NR	β-blockers
Pongwecharak 2009 Thailand[149]	Original article	RC	Adults with T2DM	OP	1630	1630	SMPC	CI	CI = 16.3	metformin

Prado 2016 Brazil[150]	Original article	PCS	Older adults (≥ 60 years)	CD	1517	333	Micromedex	DDI	NR	metformin, captopril, glibenclamide, insulin aspirin, HCT, propranolol, atenolol
Ramachandran 2020 Malaysia[151]	Original article	RCS	Adults with T2DM, on metformin with eGFR < 60 ml/ min/1.73m^2	OP	143	143	NR	CI DP	CI = 4.9 DP = 2.8	metformin
Rigler 2005 USA[152]	Original article	RCS	Older adults (≥ 60 years), Medicaid recipients	OP/ NH/ CD	3185	618	Beers Criteria (1997)	CI	CI = 8.4	amitriptyline
Romley 2015 USA[153]	Original article	RC	Older adults with DM (≥ 65 years) on glipizide or glimepiride	IP/OP	465 918	465 918	Lexicomp online, Micromedex	DDI	NR	warfarin, glipizide, glimepiride
Ruiz-Tamayo 2016 Spain[154]	Original article	RCS	Adults with T2DM on non-insulin ADM	OP	255499	255499	SMPC, international expert consensus documents, clinical practice guidelines	CI	NR	metformin, sulfonylureas, pioglitazone
Salgueiro 2018 Spain[155]	Review	NR	Older adults	IP/OP/N H	19 studies	NR	STOPP/START Criteria	PO	NR	statins
Samardzic 2015 Croatia[156]	Original article	RCS	Adults with DM on at least one ADM	OP	225	225	Lexi-Interact™ software	DDI	DDI = 80.9	metformin, HCT, insulin, glimepiride, gliclazide glyburide, sitagliptin, bisoprolol, atorvastatin, furosemide, losartan

Sankar 2015 India[157]	Original article	PCS	Prescriptions for adults with DM admitted to hospital	IP	50	50	Medscape, Drugs.com and standard reference textbooks	DDI	DDI = 70 of prescriptions	aspirin, clopidogrel, digoxin, glibenclamide, metformin, insulin
Sato 2018 Japan[158]	Original article	Case crossover	Older adults (≥ 65 years), unscheduled admissions	IP	247897	85325	Japanese Guidelines for Medical Treatment and Its Safety in the Elderly, a screening tool for PIM use in Japan	IDS	IDS = 29	glimepiride, sitagliptin, metformin
Schindler 2020 Germany[159]	Original article	Interv	Older adults with T2DM (≥ 65 years)	NR	121	121	drug interaction module of the German ABDA database	DDI DP IDS PO UDT	DP = 49.3 IDS = 1.0 PO = 3.1 UDT = 3.4 of DRPs	NR
Scotton 2009 USA[160]	Original article	Interv	Adults with T2DM receiving at least 2 doses of metformin	IP	283	283	FDA guidelines	CI	CI = 30.7	metformin
Secoli 2010 Brazil[161]	Original article	PCS	Older adults (≥ 60 years)	CD	2143	288	Micromedex	DDI	DDI = 64.6	NR
Shah 2016 Saudi Arabia[162]	Case report	CS	80 years old male with DM	IP	1	1	NICE guidelines for HTN	PO UDT	NR	spironolactone, furosemide, aspirin, clopidogrel, rosuvastatin, atorvastatin
Shareef 2015 India[163]	Original article	PCS	Adults with DM and HTN	IP	174	174	Beers Criteria, Lexicomp drug information handbook and Micromedex	DDI DP IDS PO UDT	DDI = 6.8 DP = 20.4 IDS = 22.4 PO = 8.8 UDT = 19 of DRPs	aspirin, clopidogrel, atorvastatin, metformin, CCBs, ACEIs/ARBs, carvedilol, amlodipine

Shareef 2016 India[164]	Original article	PCS	Adults with DM on at least one ADM	IP	174	174	Lexicomp drug information handbook, British National Formulary and Micromedex	DDI<br>DP<br>IDS<br>PO<br>UDT	DDI = 5.8<br>DP = 17.9<br>IDS = 16.4<br>PO = 10.6<br>UDT = 18<br>of DRPs	amlodipine, ACEIs/ARBs, metformin, amitriptyline, aspirin, clopidogrel, simvastatin, antihypertensives
Sharma 2020 India[165]	Original article	CS	Older adults with T2DM (≥ 65 years)	IP	150	150	Beers Criteria (2019)	CI<br>DP	PIP = 74	insulin, glimepiride, amitriptyline, furosemide, spironolactone
Silvestre 2007 Portugal[166]	Case report	CS	Older adults with severe lactic acidosis (> 65 years)	IP	2	2	FDA dosing recommendation for metformin	DP	NR	metformin
Simons 2014 Australia[167]	Original article	RCS	Adults with high coronary risk	NR	276 212	67595	National Heart Foundation of Australia & Cardiac Society of Australia and New Zealand- an expert guide to clinical practice for secondary prevention of CHD (2012); National Vascular Disease Prevention Alliance (2012)	PO	PO = 25	lipid-lowering drugs
Siripala 2019 Sri Lanka[168]	Original article	RCS	Older adults (≥ 60 years) on long-term medicines	CD<br>OP<br>IP	468	208	STOPP/START criteria (version 1)	CI<br>PO	CI = 9.1<br>PO = 67.3	metformin, glibenclamide, statins, β-blockers, ACEIs, antiplatelet agents,

Soorapan<br>2002<br>UK[169]	Thesis	Interv	Adults with T2DM<br>on at least one OHA	OP	198	198	NR	CI<br>DP<br>IDS<br>PO<br>UDT	CI = 0.3<br>DP = 9.8<br>IDS = 37<br>PO = 16<br>UDT = 5.2<br>of DRPs	gliclazide, metformin
Spanopoulos<br>2018<br>UK[170]	Original article	CS	Adults with T2DM,<br>initiated on a DPP-4 inhibitor	NR	3837	3837	SMPC for DPP-4 inhibitors	DP	DP = 16<br>of patients with CrCl <50	DPP-4 inhibitors (sitagliptin, saxagliptin, alogliptin, vildagliptin)
Stewart<br>2015<br>USA[171]	Original article	PCS	Adults, uninsured, using free medication reconciliation services	OP	1842	NR	NR	DDI<br>IDS<br>PO<br>UDT	NR	NR
Sulkin<br>1997<br>UK[172]	Original article	RCS	Adults with T2DM	OP	89	89	Current prescribing recommendations – literature based	CI	NR	metformin
Swanoski<br>2017<br>USA[173]	Original article	RCS	Older adults with arthritis, depression, and/or DM<br>(≥ 65 years)	OP	135381<br>681	328124<br>37	Beers criteria (2012), clinical practice guidelines, ADA guideline (2012)	IDS	IDS = 8.3	NR
Sweileh<br>2007<br>Palestine[174]	Original article	RCS	Adults with T2DM	OP	272	272	SMPC & current literature	CI	CI = 60<br>of metformin users	metformin
Tahir<br>2015<br>Indonesia[175]	Original article	PCS	Adults with T2DM hospitalised for ≥ 3 days	IP	100	100	NR	DDI<br>DP<br>PO	DDI = 17<br>DP = 58<br>PO = 24	metformin
Taner<br>2018<br>Turkey[176]	Original article	RCS	Adults with T2DM<br>on metformin	IP	66	66	NICE guideline	CI	CI = 3	metformin

Terán-Álvarez<br>2014<br>Spain[177]	Original article	RCS	Older adults<br>(> 64 years)<br>poly-medicated<br>(> 10 drugs)	OP	349	156	STOPP criteria	CI	CI = 3.8	glyburide,<br>chlorpropamide
Tirkkonen<br>2010<br>Finland[178]	Original article	RCS	Adults with T2DM on sulphonylureas	IP	3884	3884	NR	DDI	DDI = 16.1	sulfonylureas (glibenclamide, glipizide, glimepiride)
Toth<br>2003<br>Canada[179]	Original article	PCS	Adults with T2DM	OP	368	368	Canadian clinical practice guidelines (1998)	PO	NR	aspirin,<br>antihyperglycemics,<br>antihypertensives, statins
Vaccaro<br>2008<br>Italy[180]	Original article	PCS	Adults with T2DM<br>(50-75 years)<br>no previous cardiovascular events	OP	2465	2465	Italian Diabetes Society Guidelines for cardiovascular prevention in diabetic patients	PO	PO = 20	antiplatelet agents, lipid-lowering agents,<br>antihypertensives
Van Roozendaal<br>2009<br>Australia[181]	Original article	RCS	Adults with T2DM and poor glycaemic control	OP	148	148	Evidence-based checklist for the detection of drug related problems in T2DM	CI<br>DDI<br>DP<br>IDS<br>PO	CI = 0.6<br>DDI = 15.1<br>DP = 5.9<br>IDS = 2.9<br>PO = 26.7<br>of DRPs	aspirin, ACEIs/ARBs,<br>sulphonylureas,<br>metformin, insulin,<br>statins, CCBs, β-blockers
Wen<br>2011<br>Taiwan[182]	Original article	RC	Prescriptions for adults with T2DM	OP	93447<br>(2001)<br>1152330<br>(2006)	93447<br>(2001)<br>1152330<br>(2006)	Contraindications listed in the SMPC of TZD	CI	CI = 9.41 (2001)<br>CI = 12.5 (2006)<br>of prescriptions	TZD
Wermeille<br>2004<br>UK[183]	Original article	PC	Community pharmacy customers with T2DM on OHA	OP	62	62	NR	DP<br>PO<br>UDT	DP = 40.9<br>PO = 43.9<br>UDT = 9.1<br>of PIP	NR
Wood<br>1999<br>Australia[184]	Original article	CS	Adults with DM	OP	629	629	The Drugs and Therapeutics Bulletin (1994)	PO	PO = 7.2	aspirin

Woodward<br>2007<br>UK[185]	Original article	RCS	Adults with DM	OP	300	300	Joint British Societies recommendations on prevention of CHD in clinical practice (1998), ADA guideline (1997)	PO	PO = 16	antiplatelet agents
Xin<br>2016<br>China[186]	Original article	Interv	Adults with DM	IP	471 (pre-I)<br>473 (post-I)	471 (pre-I)<br>473 (post-I)	National pharmacotherapy guidelines (New Materia Medic, 2010) and local-evidence-based pharmacotherapy protocols	DDI<br>DP<br>IDS<br>PO<br>UDT	pre-intervention<br>DDI = 5.1<br>DP = 56.1<br>IDS = 6.8<br>PO = 7.4<br>UDT = 9.7<br>Post-intervention<br>DDI = 3.7<br>DP = 35.3<br>IDS = 8.9<br>PO = 6.3<br>UDT = 12.1 of medication errors	NR
Yeste-Gómez<br>2014<br>Spain[187]	Original article	PCS	Older adults (≥65 years) admitted to an internal medicine unit	IP	131	NR	STOPP/START Criteria	CI<br>PO	NR	ACEIs/ARBs, antiplatelet agents, statins
Yimama<br>2018<br>Ethiopia[188]	Original article	PCS	Adults with T2DM and HTN	OP	300	300	Pharmacotherapy text book and Ethiopian Standard Treatment Guideline	CI<br>DP<br>IDS<br>PO<br>UDT	CI = 1.2<br>DP = 17.6<br>IDS = 27.9<br>PO = 29.4<br>UDT = 10.3<br>of DRPs	NR

Zanatta 2020 Brazil[189]	Original article	CS	Adults with DM	CD	42	42	Online drug interaction checker	DDI	NR	HCT, metformin, simvastatin, aspirin
Zazuli 2017 Indonesia[190]	Original article	PCS	Adults with T2DM and HTN, on at least one ADM and anti-hypertensive agent	IP	90	90	Standards of Medical Care in Diabetes (2015), Consensus Guidelines on the Management and Prevention of T2DM in Indonesia (2015), Drug Information Handbook 2011-2012 (20th ed)	CI<br>DDI<br>DP<br>IDS<br>PO<br>UDT	CI = 1.5<br>DDI = 18<br>DP = 10.4<br>IDS = 11.5<br>PO = 25.7<br>UDT = 16.9<br>of DRPs	NR

[a] percentages are calculated from people with DM unless otherwise specified

[b] Smits KP, Sidorenkov G, Bilo HJ, Bouma M, van Ittersum FJ, Voorham J, Navis G, Denig P. Development and initial validation of prescribing quality indicators for patients with chronic kidney disease. Nephrology Dialysis Transplantation. 2016 Nov 1;31(11):1876-86

Abbreviations

AACE = American Association of Clinical Endocrinologists
ACEIs = angiotensin converting enzyme inhibitors
ACOVE-3 = Assessing Care of Vulnerable Elders-3
ADA = American Diabetes Association
ADM = antidiabetic medicine
AmMH = American Medicine handbook
AuMH = Australian Medicines Handbook
ARBs = angiotensin II receptor blockers
BNF = British National Formulary
C = control group
CD = Community Dwelling
CHD = coronary heart disease
CI = contraindication
CKD = chronic kidney disease
CrCl = creatinine clearance
CS = cross-sectional
CVD = cardiovascular disease
DDI = drug-drug interaction
DKA = diabetic ketoacidosis
DM = diabetes mellitus
DP = dosing problem
DPP-4 = Dipeptidyl peptidase 4
DRP = drug related problem
FDA = Food and Drug Administration
HCT = hydrochlorothiazide
IDS = inappropriate drug selection
I = intervention
Interv = interventional
IP = inpatient
JNC = Joint National Committee
MAI = Medication Appropriateness Index
MAT = Medication Assessment Tool
MIMS = Monthly Index of Medical Specialties
NCEP-ATP III = National Cholesterol Education Program-Adult Treatment Panel III
NH = Nursing Home
NICE = National Institute for Health and Care Excellence
NIDDM = non-insulin dependent diabetes mellitus
NKF KDOQI = National Kidney Foundation Kidney Disease Outcomes Quality Initiative
NR = not reported
OHA = oral hypoglycaemic agents
OP = outpatient
PC = prospective cohort
PCS = prospective crossectional
DPN = diabetic peripheral neuropathy
PO = prescribing omission
PQI = Prescribing quality indicator
RC = retrospective cohort
RCS = retrospective cross-sectional
SEMDSA = Society for Endocrinology, Metabolism and Diabetes of South Africa
SMPC = summary of medicinal product characteristics
SSED = Swiss Society of Endocrinology and Diabetology
START = Screening Tool to Alert to Right Treatment
STOPP = Screening Tool of Older Persons' Prescriptions
T2DM = type 2 diabetes mellitus
TZD = thiazolidinediones
UDT = unnecessary drug therapy

Supplementary Table 3: Specific PIP events reported in included studies

Author/Year/ Country	Specific PIP events for DM
Abdulmalik 2019 Ethiopia[1]	NR
Abu Farha 2019 Jordan[2]	Not prescribing ACEIs/ARBs for diabetic patient with uncontrolled blood pressure; prescribing glyburide for patients with CrCl < 50 mL/min
Ahmad 2014 Netherlands[3]	Prescribing glibenclamide for patients with frequent occurrence of hypoglycaemic episodes
Ahmed 2020 Ethiopia[4]	NR
Aketchi 2014 Kenya[5]	For T2DM patients – prescribing a long-acting sulfonylurea for elderly patients; not prescribing statins for patients with high cardiovascular risk; not prescribing aspirin for patients with history of CVD; not prescribing metformin as a first-choice drug or for overweight patients; not prescribing β-blockers for patients with hypertension and history of IHD or MI; not prescribing ACEIs/ARBs for patients with albuminuria
Al Aqqad 2014 Malaysia[6]	Prescribing glibenclamide or chlorpropamide to older adults with T2DM; prescribing β-blockers in those with DM and frequent hypoglycaemic episodes i.e. one episode per month
Alauddin 2020 USA[8]	Prescribing metformin for patients with eGFR < 30 mL/min/1.73 m^2
Al Khaja 2018 Bahrain[7]	Prescribing glyburide in older T2DM patients
Ali 2015 Pakistan[9]	NR
AL-Musawe 2020 Portugal[10]	DDI--ACEIs + ARBs; aspirin + SSRI/metimazole; CCBs + clopidogrel; digoxin + HCT/verapamil/ amiodarone/warfarin; simvastatin + warfarin; lisinopril + KCl; pravastatin + darunavir; prescribing long-acting sulfonylureas (glibenclamide or glimepiride) for older adults
Al-Taani 2017 Jordan[11]	NR
Alyazeedi 2019 Qatar[12]	Prescribing glibenclamide, insulin aspart, lispro and regular insulin for older diabetic patients
Anderson S 2020 USA[13]	Not prescribing of statins for eligible patients
Anderson T 2020 USA[14]	Prescribing intensified antidiabetic medication for patients with limited life expectancy or already at goal HbA1c
Andreassen 2016 UK[15]	NR
Arafath 2015 India[16]	DDI--glipizide + aspirin/ciprofloxacin/enalapril; insulin regular human + ciprofloxacin; metformin + nifedipine; pioglitazone + ciprofloxacin
Araújo 2013 Brazil[17]	DDI--antidiabetics + diuretics/ACEIs/anti-lipidaemic drugs/corticoids
Araújo 2019 Brazil[18]	NR
Ayele 2018 Ethiopia[19]	Prescribing single antihypertensive agent for stage II hypertensive patients; not prescribing antihypertensives for hypertensive patient; prescribing dual antihypertensive agents for stage I hypertensive patients
Babu2016 India[20]	DDI--insulin + metformin/aspirin

Basheti 2017 UAE[21]	NR
Belaiche 2012 France[22]	NR
Bezabhe 2020 Australia[23]	Prescribing metformin for adults with GFR < 30 mL/1.73m^2
Blundell 2015 Spain[24]	Prescribing glyburide or chlorpropamide in T2DM (risk of prolonged hypoglycaemia); prescribing β-blockers in diabetic patients with frequent episodes of hypoglycaemia (≥ 1 episode per month); not prescribing metformin in T2DM ± metabolic syndrome (in the absence of renal failure); not prescribing ACEIs/ARBs in diabetic nephropathy (or microalbuminuria proteinuria (> 30 mg/24h) ± renal failure); not prescribing antiplatelet and or statin therapy with coexisting cardiovascular risk factors
Borges 2010 Brazil[25]	NR
Breuker 2017 France[26]	Threefold increase in the dose of hypoglycaemic sulfamide at discharge in patient with imbalanced diabetes and renal failure; Prescribing insulin glargine instead of insulin detemir at the same dose in patient with imbalanced diabetes
Bulatova 2007 Jordan[27]	Not prescribing antiplatelet therapy for adult diabetic individuals either with history of cardiovascular disease or those at high risk
Calabrese 2002 USA[28]	Prescribing metformin despite elevated SCr concentration, pH < 7.35, or use of contrast dye
Capafons 2005 Spain[29]	Prescribing metformin for patients with creatinine values > 0.132 mmol/L
Casparie 1985 Netherlands[30]	Prescribing too much insulin dose, or improper combination of short, intermediate, or long-acting insulin in the morning or evening
Castro-Ríos 2008 Mexico[31]	Prescribing only glibenclamide for obese DM patients; incorrect dose and interval of glibenclamide and metformin
Caughey 2010 Australia[32]	Prescribing metformin for those aged 85 years old or above; prescribing long-acting sulfonylureas (gilbenclamide and glimepiride) to the elderly; prescribing TZDs in patients with moderate to severe HF; prescribing β-blockers in patients with chronic airways disease
Caughey 2017 Australia[33]	Prescribing long-acting sulphonylureas (glibenclamide, glimepiride) or α_1-blockers for older patients
Chin 2015 Pakistan[34]	DDI--insulin + ciprofloxacin/moxifloxacin/bisoprolol; duloxetine + diclofenac; aspirin + lisinopril; metformin + ciprofloxacin/cimetidine
Chou 2013 Taiwan[35]	Prescribed drug was fragmented despite being a special oral formulation
Christiaens 2020 Belgium[36]	Not adjusting antidiabetic prescription while HbA1c value is higher or lower than the patient's target range
Chung 2017 Hong Kong[37]	Metformin prescribed for a patient with increased serum creatinine level
Courtenay 2007 UK[38]	Insulin/oral medication dose not adjusted when persistent BG > 14 mmol or BG < 4 mmol; not prescribing insulin after hypoglycaemia
De Arau´jo 2020 Brazil[39]	Prescribing glibenclamide or glimepiride for older adults
Demoz 2019 Ethiopia[40]	NR
Devalia 2010 UK[41]	Incorrect insulin prescription, incorrect sliding scale
Devetzis 2011 Greece[42]	Prescribing of metformin in the presence of AKI/CKD

Diab 2012<br>Qatar[43]	Prescribing metformin at GFR < 45 mL/min; prescribing insulin before initiating on oral agents; prescribing pioglitazone for a patient having osteoporosis; not prescribing ACEIs/ARBs for a patient with microalbuminuria or proteinuria or hypertension; not prescribing tricyclic antidepressant for patients with diabetic neuropathy; not prescribing statins for patients older than 40 years; not prescribing fibrates for TG level > 4.5 mmol/L; DDI--simvastatin/atorvastatin + macrolide antibiotics/ketoconazole/itraconazole
Dias 2019<br>Brazil[44]	DDI insulin + ciprofloxacin/losartan/metformin/enalapril/carvedilol/captopril/ atenolol; metformin + atenolol/enalapril/carvedilol
Dinesh 2007<br>Nepal[45]	DDI--metformin + enalapril/ranitidine/atenolol; aspirin + enalapril/glibenclamide/ insulin; atenolol + amlodipine/gliclazide/glibenclamide
Doellner 2017<br>USA[46]	Not prescribing statins for primary prevention in patients with diabetes; use of high-risk medication in elderly patients
Dongre 2019<br>India[47]	DDIs--metformin + salbutamol/ranitidine/furosemide/clarithromycin/spironolactone/ levothyroxine/moxifloxacin; Glimepiride + aspirin/salbutamol/metoprolol
Dosa 2013<br>USA[48]	NR
Khamaisi 2012<br>Israel[91]	DDI--repaglinide + brotizolam causes severe hypoglycaemia
Ellis 2015<br>USA[49]	Major DDIs--pregabalin + naproxen; duloxetine + tramadol/metoclopramide
Elnaem 2017<br>Malaysia[50]	Not prescribing statins or prescribing non-statin therapy in statin eligible patients; DDI--amlodipine + simvastatin
Elnaem 2019 (a)<br>Malaysia[51]	Not prescribing statins for eligible patients
Elnaem 2019 (b)<br>Malaysia[52]	No statin therapy prescription for eligible patients; non-statin therapy in statin-eligible patients; low-intensity statin in high CVD risk patients; renal dose adjustment not done for the prescribed statin regimen
Emslie-Smith 2001<br>UK[53]	Metformin prescribed to patients with acute myocardial infarction, cardiac failure, renal impairment, chronic liver disease, or lactic acidosis
Erah 2013<br>Nigeria[54]	Prescribing chlorpropamide or glibenclamide (glyburide) for older DM patients; not considering dosage reduction for older adults
Faragon 2003<br>USA[55]	Not prescribing aspirin for eligible patients
Formiga 2016<br>Spain[56]	Prescribing digoxin in a dose exceeding 0.125 mg/d except for treating atrial arrhythmias; prescribing amiodarone for older patients; not prescribing ACEIs for patients with chronic HF; prescribing statins for patients with a documented history of coronary, cerebral or peripheral vascular disease, where the patient's functional status remains independent for activities of daily living and life expectancy is > 5 years; not prescribing an ACEIs or ARBs for elderly with HTN with a history of HF, left ventricular hypertrophy, IHD, chronic kidney disease, or cardiovascular accident
Frankenthal 2015<br>Israel[57]	Prescribing glibenclamide in older patients with T2DM; prescribing β-blockers in patients with frequent hypoglycaemic episode; not prescribing metformin in patients with T2DM with or without metabolic syndrome; not prescribing ACEIs or ARBs in patients with diabetes and nephropathy; not prescribing antiplatelet and/or statin therapy in patients with DM and coexisting major cardiovascular risk factors
Fu 2011<br>USA[58]	Not prescribing statins for eligible patients
Gagnon 2020<br>Canada[59]	Prescribing drugs in the Beers list (2015) for older adults

Galván-Banqueri 2013 Spain[60]	Not prescribing statins and antiplatelet agents in DM with one or more coexisting risk factors
Garat 2010 France[61]	Prescribing metformin for patients with dehydration, heart failure, liver cirrhosis, alcoholism (acute or chronic), chronic renal failure, respiratory failure or injection of iodinated contrast agent
Giorda 2020 Italy[62]	Prescribing sulphonylureas for adults aged ≥ 75 years, history of HF, unstable angina, CHD, stroke, or MI, chronic renal insufficiency (moderate to severe), history of severe hypoglycaemia, obesity, cognitive impairment, and risky occupation (bus/taxi/train driver, working at height)
Gor 2019 USA[63]	Prescribing metformin for SCr ≥ 1.4 mg/dL for women and ≥ 1.5 mg/dL for men or eGFR < 30 mL/min/1.73 m^2; prescribing glyburide for all participants with an eGFR of < 60 mL/min/1.73 m^2
Granas 2010 Norway[64]	NR
Haggerty 2005 USA[65]	Not prescribing aspirin for eligible patients
Harder 2009 Germany[66]	Not prescribing β-blockers or ACEIs/ARBs for patients with MI or CHF, not prescribing antiplatelet for TIA/stroke, not prescribing aspirin for hypertensive diabetic patients
Hartuti 2019 Indonesia[67]	Prescribing rapid-acting insulin as monotherapy; prescribing low dose of insulin, ineffective combination of insulin and metformin;<br>DDI-- antidiabetic drugs + other drugs that have a hypoglycaemic effect
Haugbølle 2006 Denmark[68]	Prescribing insulin for patients who need tablet treatment
Herman 2016 Indonesia[69]	Prescribing glibenclamide to older T2DM patients
Hinds 2016 USA[70]	Prescribing inappropriate intensity statin
Holstein 1999 Germany[71]	Prescribing metformin to patients with renal impairment, heart failure, respiratory insufficiency, or hepatic impairment
Hong 2020 South Korea[72]	Prescribing inappropriate dose of DPP-4 inhibitors for adults with CKD
Huang W 2014 Australia[74]	Prescribing metformin in the presence of cardiac failure (moderate to severe), liver dysfunction, renal dysfunction (GFR < 30), recent MI, gangrene, pancreatitis, or dehydration, respiratory failure, diabetic ketoacidosis
Huang D 2014 USA[73]	Prescribing metformin for patients with GFR < 60 mL/min/1.73 m^2
Huang W 2015a Australia[75]	Prescribing metformin for patients with renal impairment, cardiac failure or circulatory collapse
Huang W 2015b Australia[76]	Prescribing metformin for patients with moderate to severe HF, hepatic dysfunction, pancreatitis and renal failure (CrCl < 30 mL/min); prescribing metformin in an excessive dosage with respect to CrCl or eGFR
Huri & Wee 2013 Malaysia[78]	Prescribing ACEIs for patients with ESRD, prescribing α-blockers such as prazosin and doxazosin as second or third addon therapies when other better alternatives were available and not contraindicated; prescribing short-acting nifedipine for elderly patients and spironolactone for patients with CrCl < 30 mL/min; prescribing aspirin to patients with CrCl of < 10 mL/min.<br>DDI--aspirin + clopidogrel, prescribing simvastatin at more than 20 mg while receiving amlodipine

Huri & Ling 2013 Malaysia[77]	DDI--simvastatin + amlodipine/fenofibrate
Ibrahim 2005 USA[79]	DDI--major interactions (furosemide + aspirin/digoxin; aspirin + coumadin; Verapamil + digoxin/atenolol)
Ikäheimo 2019 Finland[80]	DDI--NSAIDs + aspirin/ACEIs/ARBs/β-blockers; warfarin + simvastatin
Izquierdo 2007 USA[81]	Prescribing metformin when SCr was greater than 1.4 mg/dl in females and 1.5 mg/dl in males; prescribing TZD in AHA class III or IV CHF
Jameson 2016 UK[82]	Prescribing GLP-1 receptor agonist monotherapy; prescribing non-recommended dual therapy; prescribing non-recommended triple therapy; and other therapy/addon to insulin
Jiang 2003 Georgia[83]	NR
Johnston 2013 USA[84]	Prescribing pregabalin for patients with dizziness, angioedema, decreased platelet count, or non-epileptic seizures; prescribing duloxetine for patients with uncontrolled hypertension, severe renal disease, slow gastric emptying, hyponatremia, urinary hesitation and/or retention, hepatic insufficiency, bipolar disorder, alcohol use, high-severity skin reaction, moderate-severity skin reaction, narrow-angle glaucoma, or non-epileptic seizures
Kara 2016 Turkey[85]	Prescribing β-blockers in DM with frequent hypoglycaemic episodes; not prescribing metformin for patients with T2DM ± metabolic syndrome; not prescribing ACEIs in DM with nephropathy; not prescribing antiplatelet and/or statin therapy in DM with cardiovascular risk
Karandikar 2013 India[86]	Not prescribing statins for diabetic patients with one or more co-existing major cardiovascular risk factor; β-blockers given to patients with DM and episodes of hypoglycaemia; not prescribing metformin for adults with T2DM ± metabolic syndrome
Kassam 2007 Canada[87]	Not prescribing antiplatelet therapy for eligible patients
Kavousi 2019 India[88]	NR
Kefale 2020 Ethiopia[89]	NR
Khalil 2018 Australia[90]	Prescribing metformin for patients with HF or lactic acidosis, history of severe hepatic impairment and DKA; prescribing of sulphonylureas for patients with a history of DKA, metabolic acidosis, treatment with bosentan, severe renal and hepatic impairment; prescribing insulin for patients with BSL < 3.9 mmol/L or hypoglycaemia; prescribing DPP-4 inhibitors for patients with hypoglycaemia and concomitant use of insulin or sulphonylurea and pancreatitis
Klinke 2004 Canada[92]	Not prescribing aspirin for eligible patients (DM + age ≥ 30)
Kochetkov 2019 Russia[93]	Prescribing long-acting sulfonylureas (glibenclamide, chlorpropamide, glimepiride) for elderly patients; prescribing loop diuretics in the absence of clinical signs of HF; not prescribing statin therapy in patients with documented history of coronary, cerebral or peripheral vascular disease, where the patients' functional status remains independent for daily activities and life expectancy is more than 5 years; not prescribing aspirin in patients with documented history of atherosclerotic coronary disease in patients with sinus rhythm; not prescribing clopidogrel for patients with a documented history of cerebral or PVD
Kolawole 2004 Nigeria[94]	Not prescribing aspirin for eligible patients
Korhonen 1979 Finland[95]	Prescribing biguanides for high risk patients as it induces lactic acidosis

Kosmalski 2012 Poland[96]	Prescribing metformin for patients having CHF, CKD, chronic respiratory failure, alcohol dependency syndrome, or drug intolerance
Kovacevic´ 2014 Serbia[97]	Prescribing glibenclamide for older adults with T2DM; not prescribing metformin for T2DM; not prescribing ACEIs in diabetes with nephropathy; not prescribing antiplatelet and/or statin therapy in patients with cardiovascular risk
Krishnarajan 2020 India[98]	DDI--glimepiride + fluconazole; Pioglitazone + Insulin Glargine
Kumar 2011 India[99]	DDI--atorvastatin + clopidogrel/digoxin;<br>aspirin + ACEIs/enoxaparin; digoxin + furosemide; insulin + thiazide/timolol
Kumar 2018 India[100]	NR
Kwong 1998 USA[101]	Prescribing phenformin to T2DM patient with renal insufficiency
Lahoz 2007 Spain[102]	Prescribing statins for illegible patients
Lamarr 2010 USA[103]	Not prescribing ACEIs/ARBs or aspirin for eligible patients
Lamine 2016 Switzerland[104]	Prescribing metformin or sulfonylureas in CKD stage ≥ 3b; prescribing GLP-1 agonist in CKD stage ≥ 4
Landi 2007 Italy[105]	NR
Langenhoven 2015 South Africa[106]	Not prescribing ACEIs for eligible DM-hypertensive patients; not prescribing statins or aspirin for eligible patients
Lati 2020 Kenya[107]	DDI--antidiabetic drugs + β-blockers, enalapril + metformin, nifedipine + metformin, CCBs + β-blockers
Laurent 2019 France[108]	NR
Laville 2018 France[109]	NR
Leitao 2006 Brazil[110]	Not prescribing aspirin for DM patients older than 40 years
Leonard 2016 USA[111]	DDI--sulfonylurea + antihyperlipidaemic cause severe hypoglycaemia
Lian 2008 UK[112]	NR
Lindblad 2005 USA[113]	Prescribing β-blockers for elderly and frail with DM
Liu 2012 Taiwan[114]	Not prescribing statins or aspirin in DM and coexisting major cardiovascular risk factors; not prescribing metformin for adults with T2DM in the absence of renal impairment; not prescribing ACEIs/ARBs for patients with chronic HF; not prescribing aspirin or clopidogrel with a documented history of atherosclerotic coronary or cerebral or PVD in patients with sinus rhythm
Lockery 2020 Australia/USA[115]	Prescribing long-acting sulfonylurea to older adults
Lopez 2014 Spain[116]	Prescribing glibenclamide or chlorpropamide to T2DM (risk of prolonged hypoglycaemia), not prescribing metformin for patients with T2DM ± metabolic syndrome (in the absence of renal impairment); not prescribing ACEIs/ARBs in diabetes with nephropathy (overt proteinuria or microalbuminuria (< 30 mg/24h)); not prescribing antiplatelet therapy in DM with coexisting major cardiovascular risk factors (HTN, hypercholesterolemia, smoking history); not prescribing statin therapy in diabetes mellitus if coexisting major cardiovascular risk factors present

Lu 2020<br>China[117]	NR
Luz 2018<br>Brazil[118]	Not prescribing metformin in cases of T2DM or metabolic syndrome in the absence of renal dysfunction; not prescribing ACEIs/ARBs in cases of diabetic nephropathy (e.g., proteinuria or microalbuminuria based on urinalysis > 30 mg/24 h) or renal dysfunction; not prescribing antiplatelet therapy in cases of DM plus one or more cardiovascular associated factor (e.g., HTN, hypercholesterolemia, history of smoking); not prescribing statin therapy in cases of DM plus one or more cardiovascular associated factor
Maciulaitis 2006<br>Lithuania[119]	Prescribing TZDs in case of liver failure or without investigation of its function; prescribing monotherapy with long-acting insulin; prescribing combined oral therapy without starting with monotherapy
Maheshwari 2019<br>India[120]	Prescribing metformin for patients with elevated SCr, CHF or contrast dye
Mahner 2018<br>Germany[121]	Prescribing unadjusted dose of sitagliptin, simvastatin and furosemide for adults with renal impairment; prescribing metformin in the presence of renal failure
Mahwi 2013<br>Iraq[122]	NR
Maidana 2017<br>Brazil[123]	NR
Manes 2006<br>Italy[124]	Prescribing aspirin for patients with a cardiovascular and/or coronary risk < 1.0 event/100 patients/year
Manley 2003<br>USA[125]	NR
Margiani 2014<br>Italy[126]	Prescribing metformin in a patient with AKI due to dehydration causes lactic acidosis
Marroquin 2012<br>Spain[127]	Prescribing glyburide or chlorpropamide in T2DM (risk of hypoglycaemia); not prescribing metformin in T2DM ± metabolic syndrome (in the absence of renal failure); not prescribing antiplatelet and/or statin therapy in DM with one or more major cardiovascular risk factors; the use of aspirin in patients without the need for secondary cardiovascular prevention
Masoudi 2003<br>USA[128]	Prescribing metformin or TZDs for diabetic patients with HF
Matsumura 2009<br>Japan[129]	Prescribing antidiabetics in the presence of contraindication
McFarland 2009<br>USA[130]	Prescribing sitagliptin in a dose not appropriate for renal function
Mechessa 2020<br>Ethiopia[131]	NR
Milligan 2011<br>UK[132]	NR
Mino-León 2018<br>Mexico[133]	Prescribing sulfonylurea in older adults;<br>DDI--glibenclamide + topiramate; sulfonylureas + trimethoprim/sulfamethoxazole; duloxetine + metoclopramide/aspirin /ciprofloxacin/anticoagulants/antiplatelet drugs/NSAIDs; spironolactone + NSAIDs; aspirin + NSAIDs
Modesto 2020<br>Brazil[134]	DDI--metformin + aspirin/enalapril/captopril
Molist-Brunet 2019<br>Spain[135]	Prescribing sulfonylureas for older adults; doses of metformin not adjusted for renal failure; prescribing gliflozins (SGLT2 inhibitors) in the presence of renal failure (glomerular filtration rate (eGFR) < 45 mL/min)
Mori 2017<br>Brazil[136]	Prescribing glibenclamide or chlorpropamide to T2DM (risk of prolonged hypoglycaemia); not prescribing metformin for patients with T2DM ± metabolic syndrome (in the absence of

	renal impairment); not prescribing ACEIs/ARBs in diabetes with nephropathy (overt proteinuria or microalbuminuria); not prescribing antiplatelet therapy in DM with coexisting major cardiovascular risk factors (HTN, hypercholesterolemia, smoking history); not prescribing statin therapy in DM with coexisting major cardiovascular risk factors
Muller 2016 France[137]	Prescribing oral antidiabetic drugs in a dose too high to renal function; prescribing a contraindicated antidiabetic drugs for adults with renal impairment
Mwita 2020 Botswana[138]	Not prescribing statins for eligible individuals (DM patients with CVD, CKD, age above 40 years, diabetes duration longer than ten years or in the presence of one or more of the following cardiovascular risk factors; HTN, cigarette smoker, family history of early CAD, and any albuminuria)
Najim 2010 Malaysia[139]	Prescribing metformin for patients with renal impairment, liver impairment, CVD, chronic respiratory diseases, chronic alcohol intake, stress, metabolic diseases, radiation contrast material
Nelson 2018 UK[140]	Prescribing excessive dose of metformin and sitagliptin; prescribing gliclazide for patient with renal impairment
Niehoff 2016 USA[141]	NR
Nuñez-Montenegro 2019 Spain[142]	NR
Ogamba 2016 Kenya[143]	Major DDI--enalapril + losartan/pregabalin, atenolol + carvedilol, furosemide + gentamicin, captopril + pregabalin, nifedipine + erythromycin, HCT + carbamazepine
Oktora 2020 Netherlands[144]	Prescribing long-acting sulfonylureas to adults aged 45 or above; prescribing aspirin >150mg/day for older adults, prescribing insulin sliding scale, high-dose digoxin or immediate release nifedipine for older adults
Pasina 2020 Italy[145]	Prescribing antiplatelet agents for illegible individuals, not prescribing antiplatelet agents for eligible individuals
Patel 2016 USA[146]	Prescribing TZDs for patients with HF; prescribing renally contraindicated antidiabetics for patients with eGFR < 30 mL/min/1.73 m^2
Penfornis 2014 France[147]	Prescribing metformin while GFR < 30
Pitkala 2002 Finland[148]	β-blockers prescribed for diabetic patients taking oral hypoglycaemics or insulin
Pongwecharak 2009 Thailand[149]	Prescribing metformin for patients with chronic liver diseases, chronic renal impairments, or cardiac failure
Prado 2016 Brazil[150]	Major DDI--digoxin + HCT/spironolactone; aspirin + warfarin; amiodarone + amlodipine/ atenolol/amitriptyline/fluoxetine/digoxin/nepheline; aspirin + warfarin; simvastatin + amlodipine/diltiazem/phenofibrate/verapamil; fluoxetine + mitriptyline/haloperidol/ diclofenac
Ramachandran 2020 Malaysia[151]	Prescribing metformin for diabetic patients with CKD stages 4 and 5; prescribing metformin above 1500 mg/d for diabetic patients with CKD stage 3a or above 1000 mg in CKD stage 3b
Rigler 2005 USA[152]	NR
Romley 2015 USA[153]	DDI--warfarin + glipizide/glimepiride
Ruiz-Tamayo 2016 Spain[154]	Prescribing metformin for patients with eGFR < 30 mL/min/1.73 m^2
Salgueiro 2018 Spain[155]	Not prescribing statin therapy in DM with coexisting major cardiovascular risk
Samardzic 2015 Croatia[156]	DDI--metformin + HCT; glimepiride + ramipril/sitagliptin/bisoprolol/ibuprofen/furosemide/ losartan/ciprofloxacine; sitagliptin + atorvastatin

Sankar 2015 India[157]	DDI--aspirin + ketorolac/enoxaparin/ lornoxicam/ diclofenac/ piroxicam/ heparin/ glimepiride/insulin; glibenclamide + diclofenac/ranitidine/hydrocortisone; glimepiride + budesonide; insulin + levofloxacin/metoprolol; metformin + budesonide
Sato 2018 Japan[158]	NR
Schindler 2020 Germany[159]	NR
Scotton 2009 USA[160]	Prescribing metformin for patients undergoing surgery, with renal insufficiency, intravenous contrast use, acute CHF, age > 80 years, acute MI
Secoli 2010 Brazil[161]	NR
Shah 2016 Saudi Arabia[162]	Prescribing spironolactone and furosemide while thiazide-like diuretic is preferred; DDI--aspirin + clopidogrel; rosuvastatin + atorvastatin
Shareef 2015 India[163]	Prescribing metformin in DM with impaired SCr (SCr = 1.8 mg/dl), prescribing CCBs instead of ACEIs/ARBs for HTN treatment, prescribing low dose carvedilol for dilated cardiomyopathy, prescribing high dose (thrice daily) metformin (sustained release preparations)
Shareef 2016 India[164]	Prescribing amlodipine instead of ACEIs/ ARBs for patients with DM and HTN; prescribing drugs categorized as high risk in the modified Beers Criteria (e.g. amitriptyline) to elderly
Sharma 2020 India[165]	Prescribing glimepiride or amitriptyline to the elderly; prescribing spironolactone while GFR < 30 mL/min
Silvestre 2007 Portugal[166]	Prescribing metformin in a dose > 2.5 g/day to older adults
Simons 2014 Australia[167]	Not prescribing lipid-lowering drugs for high coronary risk DM patients
Siripala 2019 Sri Lanka[168]	Prescribing glibenclamide or chlorpropamide for patients with T2DM; prescribing β-blockers in those with DM and frequent hypoglycaemic episodes; not prescribing metformin for patients with T2DM ± metabolic syndrome (in the absence of renal impairment); not prescribing ACEIs/ARBs in diabetes with nephropathy; not prescribing antiplatelet therapy in DM with coexisting major cardiovascular risk factors, not prescribing statin therapy in DM with coexisting major cardiovascular risk factors
Soorapan 2002 UK[169]	Not prescribing antihypertensive for adults with HTN; inappropriate prescribing of furosemide for the management of hypertension; not prescribing aspirin for T2DM patients with CHD or high risk for CHD
Spanopoulos 2018 UK[170]	NR
Stewart 2015 USA[171]	NR
Sulkin 1997 UK[172]	Prescribing metformin to patients with renal impairment, cardiac failure, liver disease, pulmonary disease, ischemic heart disease, peripheral vascular disease or proteinuria
Swanoski 2017 USA[173]	NR
Sweileh 2007 Palestine[174]	Prescribing metformin for patients with CHF, renal impairment, CAD, hepatic and respiratory diseases
Tahir 2015 Indonesia[175]	DDI--glibenclamide + Simvastatin/antacid, lisinopril + glimepiride/furosemide,
Taner 2018 Turkey[176]	Prescribing metformin for patients with GFR < 30 mL/min/1.73 m^2
Terán-Álvarez 2014 Spain[177]	Prescribing glibenclamide or chlorpropamide for adults with T2DM

Tirkkonen 2010<br>Finland[178]	DDI--sulfonylureas + CYP2C9-inhibitors
Toth 2003<br>Canada[179]	Not prescribing aspirin for eligible patients
Vaccaro 2008<br>Italy[180]	Not prescribing antiplatelet agents for patients with absolute cardiovascular risk; not prescribing lipid-lowering and antihypertensive medications for eligible individuals
Van Roozendaal 2009<br>Australia[181]	Not prescribing antiplatelet therapy for patients at increased cardiovascular risk; DDI--ACEIs + sulphonylurea/insulin, aspirin + NSAIDs, atorvastatin + macrolide antibiotic/ simvastatin + macrolide antibiotic, thiazide diuretic + ACEIs/ARBs + NSAIDs, ACEIs + ARBs
Wen 2011<br>Taiwan[182]	Prescribing TZD for < 18 years old, for patient with history of T1DM, severe HF, hepatic insufficiency, concomitantly with metformin in the presence of renal inefficiency, or pregnancy
Wermeille 2004<br>UK[183]	NR
Wood 1999<br>Australia[184]	Not prescribing aspirin for patients with macrovascular disease and no contraindication
Woodward 2007<br>UK[185]	Not prescribing antiplatelet therapy to diabetic patients with established large-vessel disease (IHD, PVD or CVD); or HTN; or nephropathy or microalbuminuria
Xin 2016<br>China[186]	DDI--warfarin + omeprazole
Yeste-Gómez 2014<br>Spain[187]	Not prescribing ACEIs/ARBs for diabetes with nephropathy, i.e. overt proteinuria in routine urine or micro-albuminuria; not prescribing antiplatelet therapy in DM with coexisting major cardiovascular risk factors (HTN, hypercholesterolemia, smoking history); not prescribing statin therapy in DM with coexisting major cardiovascular risk factors
Yimama 2018<br>Ethiopia[188]	Not prescribing statins for eligible patients
Zanatta 2020<br>Brazil[189]	DDI--metformin + HCT/simvastatin/aspirin/ paracetamol, aspirin + simvastatin, simvastatin + amlodipine, atenolol + clonidine, losartan + spironolactone
Zazuli 2017<br>Indonesia[190]	NR

Abbreviations

AHA = American Heart Association
ACEIs = angiotensin converting enzyme inhibitors
AKI = acute kidney injury
ARBs = angiotensin II receptor blockers
BG = blood glucose
CAD = coronary arterial disease
CCBs = calcium channel blockers
CHD = coronary heart disease
CHF = congested heart failure
CKD = chronic kidney disease
CrCl = creatinine clearance
CVD = cardiovascular disease
Cyp2C9 = cytochrome 2C9
DDI = drug-drug interaction
DKA = diabetic ketoacidosis
DM = diabetes mellitus
DPP = dipeptidyl peptidase
eGFR = estimated glomerular filtration rate
ESRD = end stage renal disease
GFR = glomerular filtration rate
GLP = Glucagon-like peptide
HbA1c = haemoglobin A1c
HCT = hydrochlorothiazide
HF = heart failure
HTN = hypertension
IHD = ischemic heart disease
KCl = potassium chloride
MI = myocardial infarction
NR = not reported
NSAIDs = non-steroidal anti-inflammatory drugs
PVD = peripheral vascular disease
SCr = serum creatinine
SGLT = sodium-glucose co-transporter
SSRI = selective serotonin reuptake inhibitor
T2DM = type 2 diabetes mellitus
TG = triglyceride
TIA = transit ischaemic attack
TZD = thiazolidinedione

References

1. Abdulmalik H, Tadiwos Y, and Legese N. Assessment of drug-related problems among type 2 diabetic patients on follow up at Hiwot Fana Specialized University Hospital, Harar, Eastern Ethiopia. BMC Res Notes. 2019;12(1):1-6 https://doi.org/10.1186/s13104-019-4760-8.
2. Abu Farha RK, Mukattash TL, Qudah R, Alkhalaileh W, and Alsaffar S. Drug-related problems and health-related quality of life in outpatients with type 2 diabetes: A cross-sectional study from Jordan. J Pharm Health Serv Res. 2019;10(3):303-9 https://doi.org/10.1111/jphs.12268.
3. Ahmad A, Mast MR, Nijpels G, Elders PJ, Dekker JM, and Hugtenburg JG. Identification of drug-related problems of elderly patients discharged from hospital. Patient Prefer Adherence. 2014;8155-65 https://doi.org/10.2147/PPA.S48357.
4. Ahmed MY, Ejigu SH, Zeleke AZ, and Hassen MY. Glycemic control, diabetes complications and their determinants among ambulatory diabetes mellitus patients in Southwest Ethiopia: A prospective cross-sectional study. Diabetes Metab Syndr Obes. 2020;13:1089-95 https://doi.org/10.2147/DMSO.S227664.
5. Aketchi IE, Quality of prescribing in type 2 diabetes ambulatory care at Webuye District Hospital, Western Kenya. 2014, University of Nairobi.
6. Al Aqqad SM, Chen LL, Shafie AA, Hassali MA, and Tangiisuran B. The use of potentially inappropriate medications and changes in quality of life among older nursing home residents. Clin Interv Aging. 2014;9201-7 https://doi.org/10.2147/CIA.S52356.
7. Alauddin T and Petite SE. Evaluation of the safety and efficacy of metformin use in hospitalized, non–critically ill patients. J Pharm Technol. 2020;36(3):102-9 https://doi.org/10.1177/8755122520911.
8. Al Khaja KA, Isa HA, Veeramuthu S, and Sequeira RP. Potentially inappropriate prescribing in older adults with hypertension or diabetes mellitus and hypertension in a primary care setting in Bahrain. Med Princ Pract. 2018;27:241-9 https://doi.org/10.1159/000488055.
9. Ali I, Khan J, Khan A, Ullah I, and Ahmad F. Pharmacotherapy evaluation of diabetic patients in ward of general medicine, North West General Hospital & Research Centre, a case study from Khyber Pakhtunkhwa, Pakistan. Pharmacol Online. 2015;1104-8.
10. AL-Musawe L, Torre C, Guerreiro JP, Rodrigues AT, Raposo JF, Mota-Filipe H, et al. Polypharmacy, potentially serious clinically relevant drug-drug interactions, and inappropriate medicines in elderly people with type 2 diabetes and their impact on quality of life. Pharmacol res perspect. 2020;8:e00621: https://doi.org/10.1002/prp2.621.
11. Al-Taani GM, Al-Azzam SI, Alzoubi KH, Elhajji FWD, Scott MG, Alfahel H, et al. Prediction of drug-related problems in diabetic outpatients in a number of hospitals, using a modeling approach. Drug Healthc Patient Saf. 2017;965-70 https://doi.org/10.2147/DHPS.S125114.
12. Alyazeedi A, Algendy AF, Sharabash M, and Karawia A. Prevalence, determinants and associated risk of potentially inappropriate prescribing for older adults in Qatar: A national retrospective study. Clin Interv Aging. 2019;141889-99 https://doi.org/10.2147/CIA.S222532.
13. Anderson SL, Marrs JC, Chachas CR, Cichon BS, Cizmic AD, Calderon BB, et al. Evaluation of a pharmacist-led intervention to improve statin use in persons with diabetes. J Manag Care Spec Pharm. 2020;26(7):910-7 https://doi.org/10.18553/jmcp.2020.26.7.910.
14. Anderson TS, Lee S, Jing B, Fung K, Ngo S, Silvestrini M, et al. Prevalence of Diabetes Medication Intensifications in Older Adults Discharged From US Veterans Health Administration Hospitals. JAMA netw open. 2020;3:201511: https://doi.org/10.1001/jamanetworkopen.2020.1511
15. Andreassen LM, Kjome RLS, Solvik UO, Houghton J, and Desborough JA. The potential for deprescribing in care home residents with Type 2 diabetes. Int J Clin Pharm. 2016;38(4):977-84 https://doi.org/10.1007/s11096-016-0323-4.

16. Arafath AYM, Khan J, Joy J, and Jeenu JP. Assessment of drug-drug interactions in diabetic patients in a secondary care hospital. Indo Am j pharm sci. 2015;2(8):1178-89.
17. de Araújo MFM, dos Santos Alves PdJ, Veras VS, de Araújo TM, Zanetti ML, and Damasceno MMC. Drug interactions in Brazilian type 2 diabetes patients. Int J Nurs Pract. 2013;19(4):423-30 https://doi.org/10.1111/ijn.12078.
18. Araújo LU, Santos DF, Bodevan EC, Cruz HLd, Souza Jd, and Silva-Barcellos NM. Patient safety in primary health care and polypharmacy: cross-sectional survey among patients with chronic diseases. Rev Lat Am Enfermagem. 2019;27 https://doi.org/10.1590/1518-8345.3123.3217.
19. Ayele Y, Melaku K, Dechasa M, Ayalew MB, and Horsa BA. Assessment of drug related problems among type 2 diabetes mellitus patients with hypertension in Hiwot Fana Specialized University Hospital, Harar, Eastern Ethiopia. BMC Res Notes. 2018;11:728: https://doi.org/10.1186/s13104-018-3838-z.
20. Babu M, R N, Kumar SR, Dr B, and Joseph J. Evaluation of prescription pattern and associated drug interactions in type 2 diabetes mellitus patients with comorbidities and complications in a teaching care hospital. Int Res J Pharm. 2016;7:48-52 https://doi.org/10.7897/2230-8407.07551.
21. Basheti IA and Abu Gharbieh E. Revealing treatment related problems for outpatients with alarming health status in the United Arab Emirates. J Pharm Health Serv Res. 2017;8(3):193-9 https://doi.org/10.1111/jphs.12186.
22. Belaiche S, Romanet T, Allenet B, Calop J, and Zaoui P. Identification of drug-related problems in ambulatory chronic kidney disease patients: A 6-month prospective study. J Nephrol. 2012;25(5):782-8.
23. Bezabhe WM, Kitsos A, Saunder T, Peterson GM, Bereznicki LR, Wimmer BC, et al. Medication prescribing quality in Australian primary care patients with chronic kidney disease. J Clin Med. 2020;9:783: https://doi.org/10.3390/jcm9030783.
24. Blundell DB. Medication review according to STOPP-START criteria in older patients from the dose administration aids service of a community pharmacy. Farmaceuticos Comunitarios. 2015;7(2):31-36.
25. Borges APdS, Guidoni CM, Ferreira LgD, Freitas Od, and Pereira LRgL. The pharmaceutical care of patients with type 2 diabetes mellitus. Pharm World Sci. 2010;32(6):730-6 https://doi.org/10.1007/s11096-010-9428-3.
26. Breuker C, Abraham O, Di Trapanie L, Mura T, Macioce V, Boegner C, et al. Patients with diabetes are at high risk of serious medication errors at hospital: Interest of clinical pharmacist intervention to improve healthcare. Eur J Intern Med. 2017;3838-45 https://doi.org/10.1016/j.ejim.2016.12.003.
27. Bulatova NR, Al Motassem FY, and AbuRuz SM. Antiplatelet therapy for primary and secondary prevention in Jordanian patients with diabetes mellitus. Thromb Res. 2007;121(1):43-50 https://doi.org/10.1016/j.thromres.2007.03.006.
28. Calabrese AT, Coley KC, DaPos SV, Swanson D, and Rao RH. Evaluation of prescribing practices: risk of lactic acidosis with metformin therapy. Arch Intern Med. 2002;162(4):434-7 https://doi.org/10.1001/archinte.162.4.434.
29. Capafons SR, Biosca MG, and Settler RP. Follow-up of the use of metformin among the high risk population. Farmacia hospitalaria. 2005;29(6):364-6 https://doi.org/10.1016/s1130-6343(05)73697-7.
30. Casparie AF and Elving LD. Severe hypoglycemia in diabetic patients: frequency, causes, prevention. Diabetes Care. 1985;8(2):141-5 https://doi.org/10.2337/diacare.8.2.141.
31. Castro-Rios A, Reyes-Morales H, and Perez-Cuevas R. An evaluation of a continuing medical education program for primary care services in the prescription of hypoglycemic agents in

diabetes mellitus type 2. Salud Publica Mex. 2008;50 Suppl 4:S445-52 https://doi.org/10.1590/s0036-36342008001000005.
32. Caughey GE, Roughead EE, Vitry AI, McDermott RA, Shakib S, and Gilbert AL. Comorbidity in the elderly with diabetes: identification of areas of potential treatment conflicts. Diabetes Res Clin Pract. 2010;87(3):385-93 https://doi.org/10.1016/j.diabres.2009.10.019.
33. Caughey G, Barratt J, Shakib S, Kemp-Casey A, and Roughead E. Medication use and potentially high-risk prescribing in older patients hospitalized for diabetes: A missed opportunity to improve care. Diabet Med. 2017;34(3):432-9 https://doi.org/10.1111/dme.13148.
34. Chin P, Hou Y, Zhai X, Zhang Z, Yu X, Murtaza G, et al. Assessment of potential drug-drug Interactions and their associated factors in hospitalised diabetic patients. Lat Am J Pharm. 2015;34(2):217-22.
35. Chou C-Y, Hsu C-C, Chiang S-C, Ho C-C, Chou C-L, Wu M-S, et al. Association between physician specialty and risk of prescribing inappropriate pill splitting. PLoS One. 2013;8e70113 https://doi.org/10.1371/journal.pone.0070113.
36. Christiaens A, Boland B, Germanidis M, Dalleur O, and Henrard S. Poor health status, inappropriate glucose-lowering therapy and high one-year mortality in geriatric patients with type 2 diabetes. BMC Geriatr. 2020;20(1):1-9 https://doi.org/10.1186/s12877-020-01780-9.
37. Chung AY, Anand S, Wong IC, Tan KC, Wong CF, Chui WC, et al. Improving medication safety and diabetes management in Hong Kong: a multidisciplinary approach. Hong Kong Med J. 2017;23(2):158-67 https://doi.org/10.12809/hkmj165014.
38. Courtenay M, Carey N, James J, Hills M, and Roland J. An evaluation of a specialist nurse prescriber on diabetes inpatient service delivery. Practical Diabetes Int. 2007;24(2):69-74 https://doi.org/10.1002/pdi.1056.
39. de Araújo NC, Silveira EA, Mota BG, Neves Mota JP, de Camargo Silva AEB, Alves Guimarães R, et al. Potentially inappropriate medications for the elderly: Incidence and impact on mortality in a cohort ten-year follow-up. PLoS One. 2020;15e0240104 https://doi.org/10.1371/journal.pone.0240104.
40. Demoz GT, Berha AB, Alebachew Woldu M, Yifter H, Shibeshi W, and Engidawork E. Drug therapy problems, medication adherence and treatment satisfaction among diabetic patients on follow-up care at Tikur Anbessa Specialized Hospital, Addis Ababa, Ethiopia. PLoS One. 2019;14e0222985 https://doi.org/10.1371/journal.pone.0222985.
41. Devalia B. Adherance to protocol during the acute management of diabetic ketoacidosis: would specialist involvement lead to better outcomes? Int J Clin Pract. 2010;64(11):1580-2 https://doi.org/10.1111/j.1742-1241.2010.02348.x.
42. Devetzis V, Passadakis P, Panagoutsos S, Theodoridis M, Thodis E, Georgoulidou A, et al. Metformin-related lactic acidosis in patients with acute kidney injury. Int Urol Nephrol. 2011;43(4):1243-8 https://doi.org/10.1007/s11255-010-9845-1.
43. Diab MI, Pharmaceutical care in management of type 2 diabetes and primary prevention of cardiovascular disease with risk analysis of developing cardiovascular events. 2012, University of Strathclyde.
44. Dias BM, Santos FSd, and Reis AMM. Potential drug interactions in drug therapy prescribed for older adults at hospital discharge: cross-sectional study. Sao Paulo Med J. 2019;137(4):369-78 https://doi.org/10.1590/1516-3180.2019.013405072019
45. Dinesh KU, Subish P, Pranaya M, Shankar PR, Anil SK, and Durga B. Pattern of potential drug-drug interactions in diabetic out-patients in a tertiary care teaching hospital in Nepal. Med J Malaysia. 2007;62(4):294-8.

46. Doellner JF, Dettloff RW, DeVuyst-Miller S, and Wenstrom KL. Prescriber acceptance rate of pharmacists' recommendations. J Am Pharm Assoc (2003). 2017;57(3):S197-S202 https://doi.org/10.1016/j.japh.2017.03.002.
47. Dongre S, Paulose A, N N, Jacob A, As-Hal S, Rouf S, et al. A study of the possible drug-drug interactions involving oral antidiabetic drugs in patients with type II diabetes. Int Res J Pharm. 2019;10:141-144 https://doi.org/10.7897/2230-8407.1005180.
48. Dosa D, Cai S, Gidmark S, Thomas K, and Intrator O. Potentially inappropriate medication use in veterans residing in community living centers: have we gotten better? J Am Geriatr Soc. 2013;61(11):1994-1999 https://doi.org/10.1111/jgs.12516.
49. Khamaisi M. Severe hypoglycaemia from repaglinide-brotizolam drug interaction: A case report and literature review. Diabet Med. 2012;29(9):1214-5 https://doi.org/10.1111/j.1464-5491.2012.03631.x.
50. Ellis JJ, Sadosky AB, Ten Eyck LL, Mudumby P, Cappelleri JC, Ndehi L, et al. A retrospective, matched cohort study of potential drug-drug interaction prevalence and opioid utilization in a diabetic peripheral neuropathy population initiated on pregabalin or duloxetine. BMC Health Serv Res. 2015;15(1):159 https://doi.org/10.1186/s12913-015-0829-9.
51. Elnaem M, Nik Mohamed M, Huri H, and Azarisman S. Patterns of statin therapy prescribing among hospitalized patients with type 2 diabetes mellitus in two Malaysian tertiary hospitals. Trop J Pharm Res. 2017;16(12):3005-11 https://doi.org/10.4314/tjpr.v16i12.27.
52. Elnaem MH, Mohamed MHN, Huri HZ, and Shah ASM. Effectiveness and prescription pattern of lipid-lowering therapy and its associated factors among patients with type 2 diabetes mellitus in Malaysian primary care settings. Ther Clin Risk Manag. 2019;15137-145 https://doi.org/10.2147/TCRM.S182716.
53. Elnaem MH, Mohamed MHN, and Huri HZ. Pharmacist-led academic detailing improves statin therapy prescribing for Malaysian patients with type 2 diabetes: quasi experimental design. PLoS One. 2019;14(9):e0220458 https://doi.org/https://doi.org/10.1371/journal.pone.0220458.
54. Emslie-Smith AM, Boyle DI, Evans J, Sullivan F, and Morris AD. Contraindications to metformin therapy in patients with type 2 diabetes-a population-based study of adherence to prescribing guidelines. Diabet Med. 2001;18(6):483-8 https://doi.org/10.1046/j.1464-5491.2001.00509.x.
55. Erah PO and Eroje HI. Prescribing of antidiabetic medicines to older diabetes type 2 patients in Lagos, Nigeria. Nig Q J Hosp Med. 2013;23(1):12-6.
56. Faragon JJ, Waite NM, Hobson EH, Seoldo N, VanAmburgh JA, and Migden H. Improving aspirin prophylaxis in a primary care diabetic population. Pharmacotherapy. 2003;23(1):73-79 https://doi.org/10.1592/phco.23.1.73.31923.
57. Formiga F, Vidal X, Agusti A, Chivite D, Roson B, Barbe J, et al. Inappropriate prescribing in elderly people with diabetes admitted to hospital. Diabet Med. 2016;33(5):655-62 https://doi.org/10.1111/dme.12894.
58. Frankenthal D, Lerman Y, and Lerman Y. The impact of hospitalization on potentially inappropriate prescribing in an acute medical geriatric division. Int J Clin Pharm. 2015;37(1):60-7 https://doi.org/10.1007/s11096-014-0040-9.
59. Fu AZ, Zhang Q, Davies MJ, Pentakota S-R, Radican L, and Seck T. Underutilization of statins in patients with type 2 diabetes in US clinical practice: a retrospective cohort study. Curr Med Res Opin. 2011;27(5):1035-40 https://doi.org/10.1185/03007995.2011.567257.
60. Gagnon M-E, Sirois C, Simard M, Roux B, and Plante C. Potentially inappropriate medications in older individuals with diabetes: a population-based study in Quebec, Canada. Prim Care Diabetes. 2020;14(5):529-37 https://doi.org/10.1016/j.pcd.2020.03.003.
61. Galvan-Banqueri M, Gonzalez-Mendez AI, Alfaro-Lara ER, Nieto-Martin MD, Perez-Guerrero C, and Santos-Ramos B. Evaluation of the appropriateness of pharmacotherapy in patients

with high comorbidity. Aten Primaria. 2013;45(5):235-43 https://doi.org/10.1016/j.aprim.2012.11.010.
62. Garat A, Tresch E, Glowacki F, Allorge D, Houdret N, Nisse P, et al. Analysis of 30 case reports of acute poisoning with metformin during long-term therapy. Acta Clin Belg. 2010;65(sup1):18-23 https://doi.org/10.1179/acb.2010.104.
63. Giorda CB, Orsi E, De Cosmo S, Bossi AC, Guerzoni C, Cercone S, et al. Prescription of Sulphonylureas among patients with type 2 diabetes mellitus in italy: results from the retrospective, observational multicentre cross-sectional SUSCIPE (Sulphonyl_UreaS_Correct_Internal_Prescription_Evaluation) Study. Diabetes Ther. 2020;11(9):2105-19 https://doi.org/10.1007/s13300-020-00871-5.
64. Gor D, Gerber BS, Walton SM, Lee TA, Nutescu EA, and Touchette DR. Antidiabetic drug use trends in patients with type 2 diabetes mellitus and chronic kidney disease: A cross-sectional analysis of the National Health and Nutrition Examination Survey. J Diabetes. 2020;12(5):385-95 https://doi.org/10.1111/1753-0407.13003.
65. Granas AG, Berg C, Hjellvik V, Haukereid C, Kronstad A, Blix HS, et al. Evaluating categorisation and clinical relevance of drug-related problems in medication reviews. Pharm World Sci. 2010;32(3):394-403 https://doi.org/10.1007/s11096-010-9385-x.
66. Haggerty SA, Cerulli J, Zeolla MM, Cottrell JS, Weck MB, and Faragon JJ. Community pharmacy target intervention program to improve aspirin use in persons with diabetes. J Am Pharm Assoc (2003). 2005;45(1):17-22 https://doi.org/10.1331/1544345052843020.
67. Harder S, Saal K, Blauth E, Beyer M, and Gerlach FM. Appropriateness and surveillance of medication in a cohort of diabetic patients on polypharmacy. Int J Clin Pharmacol Ther. 2009;47(2):104-10 https://doi.org/10.5414/cpp47104.
68. Hartuti S, Nasution A, and Syafril S. The effect of drug-related problems on blood glucose level in the treatment of patients with type 2 diabetes mellitus. Open Access Maced. J. Med. Sci. 2019;7(11):1798-802 https://doi.org/10.3889/oamjms.2019.290.
69. Haugbølle LS and Sørensen EW. Drug-related problems in patients with angina pectoris, type 2 diabetes and asthma interviewing patients at home. Pharm World Sci. 2006;28(4):239-47 https://doi.org/10.1007/s11096-006-9023-9.
70. Herman H, San IP, Ningsih R, and Azizah RN. Inappropriate use of the drug to elderly patients with type-II diabetes mellitus in Makassar, Indonesia. Der Pharmacia Lettre. 2016;8:154-8.
71. Hinds A, Lopez D, Rascati K, Jokerst J, and Srinivasa M. Adherence to the 2013 blood cholesterol guidelines in patients with diabetes at a PCMH: comparison of physician only and combination physician/pharmacist visits. The Diabetes Educator. 2016;42(2):228-33 https://doi.org/10.1177/0145721716631431.
72. Holstein A, Nahrwold D, Hinze S, and Egberts EH. Contraindications to metformin therapy are largely disregarded. Diabet Med. 1999;16(8):692-6 https://doi.org/10.1046/j.1464-5491.1999.00115.x.
73. Hong S, Han K, and Park C-Y. Outcomes for inappropriate renal dose adjustment of dipeptidyl peptidase-4 inhibitors in patients with type 2 diabetes mellitus: Population-based study. Mayo Clin Proc. 2020;95(1):101-12 https://doi.org/10.1016/j.mayocp.2019.06.010.
74. Huang W, Castelino R, and Peterson G. Metformin usage in type 2 diabetes mellitus: Are safety guidelines adhered to? Intern Med J. 2014;44(3):266-72 https://doi.org/10.1111/imj.12369.
75. Huang DL, Abrass IB, and Young BA. Medication safety and chronic kidney disease in older adults prescribed metformin: A cross-sectional analysis. BMC Nephrol. 2014;15(1):86 https://doi.org/10.1186/1471-2369-15-86.
76. Huang W, Castelino RL, and Peterson GM. Adverse event notifications implicating metformin with lactic acidosis in Australia. J Diabetes Complications. 2015;29(8):1261-5 https://doi.org/10.1016/j.jdiacomp.2015.06.001.

77. Huang W, Peterson GM, Zaidi STR, and Castelino RL. Metformin utilisation in Australian community and aged care settings. Diabetes Res Clin Pract. 2015;108(2):336-41 https://doi.org/10.1016/j.diabres.2015.01.041.
78. Huri HZ and Wee HF. Drug related problems in type 2 diabetes patients with hypertension: a cross-sectional retrospective study. BMC Endocr Disord. 2013;13(1):2 https://doi.org/10.1186/1472-6823-13-2.
79. Huri HZ and Ling LC. Drug-related problems in type 2 diabetes mellitus patients with dyslipidemia. BMC Public Health. 2013;13(1):1192 https://doi.org/10.1186/1471-2458-13-1192.
80. Ibrahim IA, Kang E, and Dansky KH. Polypharmacy and possible drug-drug interactions among diabetic patients receiving home health care services. Home Health Care Serv Q. 2005;24(1-2):87-99 https://doi.org/10.1300/J027v24n01_07.
81. Ikäheimo I, Karjalainen M, Tiihonen M, Haanpää M, Kautiainen H, Saltevo J, et al. Clinically relevant drug-drug interactions and the risk for drug adverse effects among home-dwelling older persons with and without type 2 diabetes. J Clin Pharm Ther. 2019;44(5):735-41 https://doi.org/10.1111/jcpt.12854.
82. Izquierdo R, Meyer S, Starren J, Goland R, Teresi J, Shea S, et al. Detection and remediation of medically urgent situations using telemedicine case management for older patients with diabetes mellitus. Ther Clin Risk Manag. 2007;3(3):485-9.
83. Jameson K, D'Oca K, Leigh P, and Murray-Thomas T. Adherence to NICE guidance on glucagon-like peptide-1 receptor agonists among patients with type 2 diabetes mellitus: an evaluation using the Clinical Practice Research Datalink. Curr Med Res Opin. 2016;32(1):49-60 https://doi.org/10.1185/03007995.2015.1101372.
84. Jiang R, Potentially inappropriate medication use in Georgia nursing homes. 2003, The University of Georgia.
85. Johnston SS, Udall M, Cappelleri JC, Johnson BH, Shrady G, Chu B-C, et al. Cost comparison of drug–drug and drug–condition interactions in patients with painful diabetic peripheral neuropathy treated with pregabalin versus duloxetine. Am J Health Syst Pharm. 2013;70(24):2207-17 https://doi.org/10.2146/ajhp130088.
86. Kara O, Arik G, Kizilarslanoglu MC, Kilic MK, Varan HD, Sumer F, et al. Potentially inappropriate prescribing according to the STOPP/START criteria for older adults. Aging Clin Exp Res. 2016;28(4):761-8 https://doi.org/10.1007/s40520-015-0475-4.
87. Karandikar Y, Chaudhari S, Dalal N, Sharma M, and Pandit V. Inappropriate prescribing in the elderly: A comparison of two validated screening tools. J Clin Gerontol Geriatr. 2013;4(4):109-14 https://doi.org/10.1016/j.jcgg.2013.04.004.
88. Kassam R and Meneilly GS. Role of the pharmacist on a multidisciplinary diabetes team. Can J Diabetes. 2007;31(3):215-22 https://doi.org/10.1016/S1499-2671(07)13008-2.
89. Kavousi S and Jayprakash G. Assessment of prescription pattern of antidiabetic drugs in the inpatient department of a tertiary care hospital. Int J Pharm Sci Rev Res. 2019;58(2):89-95.
90. Kefale B, Tegegne GT, Kefale Y, Molla M, Ewunetei A, and Degu A. Magnitude and determinants of drug therapy problems among type 2 diabetes mellitus patients with hypertension in Ethiopia. SAGE Open Med. 2020;8:1-8 https://doi.org/10.1177/2050312120954695.
91. Khalil V, Sajan C, Tsai T, and Ma D. Antidiabetics usage in type 2 diabetes mellitus: are prescribing guidelines adhered to? A single centre study. Diabetes Metab Syndr. 2018;12(5):635-41 https://doi.org/10.1016/j.dsx.2018.04.005.
92. Klinke JA, Johnson JA, Guirguis LM, Toth EL, Lee T, Lewanczuk RZ, et al. Underuse of aspirin in type 2 diabetes mellitus: Prevalence and correlates of therapy in rural Canada. Clin Ther. 2004;26(3):439-46 https://doi.org/10.1016/S0149-2918(04)90040-9.

93. Kochetkov AI, De VA, Voevodina NY, Chachiashvili MV, Grishina AV, and Ostroumova OD. The application of the STOPP/START criteria in the elderly patients with type 2 diabetes mellitus and essential hypertension at the endocrinology department of a multidisciplinary hospital. Arterial Hypertension. 2019;25(3):214-24 https://doi.org/10.18705/1607-419X-2019-25-3-214-224.
94. Kolawole B, Adebayo R, and Aloba O. An assessment of aspirin use in a Nigerian diabetes outpatient clinic. Niger J Med. 2004;13(4):405-6.
95. Korhonen T, Idanpaan-Heikkila J, and Aro A. Biguanide-induced lactic acidosis in Finland. Eur J Clin Pharmacol. 1979;15(6):407-10 https://doi.org/10.1007/BF00561739.
96. Kosmalski M, Drozdowska A, Sliwinska A, and Drzewoski J. Inappropriate metformin prescribing in elderly type 2 diabetes mellitus (T2DM) patients. Adv Med Sci. 2012;57(1):65-70 https://doi.org/10.2478/v10039-012-0017-7.
97. Kovačević SV, Simišić M, Rudinski SS, Ćulafić M, Vučićević K, Prostran M, et al. Potentially inappropriate prescribing in older primary care patients. PLoS One. 2014;9(4):e95536 https://doi.org/10.1371/journal.pone.0095536.
98. Krishnarajan D, Ranjini B, S A, and Sunny S. Prescription analysis, drug interaction checking and impact of patient counseling by a clinical pharmacist in type II diabetes mellitus patients in a tertiary care hospital-a prospective study. Int J Pharm Pharm Sci. 2020;12(7):59-65 https://doi.org/10.22159/ijpps.2020v12i7.36213.
99. Kumar MA, Nizar A, Shailaja K, Jayasutha J, and Ramasamy C. Study on prescribing pattern and potentials drug-drug interactions in type-2 diabetes mellitus (inpatients) in a tertiary care teaching hospitals. Der Pharmacia Lettre. 2011;3(4):13-9.
100. Kumar S, Abhinand C, Harsh PA, and Antin S. Assessment of drug related problems in type 2 diabetes patients with hypertension: A prospective study. MJPS. 2018;4(2):14-8.
101. Kwong SC and Brubacher J. Phenformin and lactic acidosis: A case report and review. J Emerg Med. 1998;16(6):881-6 https://doi.org/10.1016/S0736-4679(98)00103-6.
102. Lahoz C, Vicente I, Criado A, Laguna F, Torrecilla E, and Mostaza J. Clinical factors associated with inappropriate prescription of statins. Med Clin (Barc). 2007;129(3):86-90 https://doi.org/10.1157/13107367.
103. LaMarr B, Valdez C, Driscoll K, and Ryan M. Influence of pharmacist intervention on prescribing of angiotensin-converting-enzyme inhibitors, angiotensin II-receptor blockers, and aspirin for diabetic patients. Am J Health Syst Pharm. 2010;67(4):290-4 https://doi.org/10.2146/ajhp090009.
104. Lamine F, Lalubin F, Pitteloud N, Burnier M, and Zanchi A. Chronic kidney disease in type 2 diabetic patients followed-up by primary care physicians in Switzerland: Prevalence and prescription of antidiabetic drugs. Swiss Med Wkly. 2016;146 https://doi.org/10.4414/smw.2016.14282.
105. Landi F, Russo A, Liperoti R, Barillaro C, Danese P, Pahor M, et al. Impact of inappropriate drug use on physical performance among a frail elderly population living in the community. Eur J Clin Pharmacol. 2007;63(8):791-9 https://doi.org/10.1007/s00228-007-0321-5.
106. Langenhoven W, Evaluating adherence to recommended clinical guidelines for the prevention of cardiovascular disease in patients with type 2 diabetes mellitus at primary care level. 2015, University of Cape Town.
107. Lati MS, David NG, and Kinuthia RN. The clinical significance of potential drug-drug interactions and their targets for minimization among hypertensive diabetic outpatients at a Kenyan referral hospital. Int J Pharm Pharm Sci. 2020;12(10):6-11 https://doi.org/10.22159/ijpps.2020v12i10.38816.
108. Laurent C, Glele LSA, Lazzarotti A, Petit JM, Vergès B, and Bouillet B. Who is at risk for drug-drug interactions in type 2 diabetic patients? Presse Med. 2019;48(12):1576-8 https://doi.org/10.1016/j.lpm.2019.09.059.

109. Laville SM, Metzger M, Stengel B, Jacquelinet C, Combe C, Fouque D, et al. Evaluation of the adequacy of drug prescriptions in patients with chronic kidney disease: results from the CKD-REIN cohort. Br J Clin Pharmacol. 2018;84(12):2811-23 https://doi.org/10.1111/bcp.13738.
110. Leitão CB, Krahe AL, Nabinger GB, Picon PX, Pecis M, Zaslavsky LM, et al. Aspirin therapy is still underutilized among patients with type 2 diabetes. Arq Bras Endocrinol Metabol. 2006;50(6):1014-9 https://doi.org/10.1590/S0004-27302006000600006.
111. Leonard CE, Bilker WB, Brensinger CM, Han X, Flory JH, Flockhart DA, et al. Severe hypoglycemia in users of sulfonylurea antidiabetic agents and antihyperlipidemics. Clin Pharmacol Ther. 2016;99(5):538-47 https://doi.org/10.1002/cpt.297.
112. Lian I, An evaluation of pharmaceutical care delivery to patients with diabetes and development of standardised assessment tools. 2008, University of Tromsø.
113. Lindblad CI, Artz MB, Pieper CF, Sloane RJ, Hajjar ER, Ruby CM, et al. Potential drug—disease interactions in frail, hospitalized elderly veterans. Ann Pharmacother. 2005;39(3):412-17.
114. Liu C-L, Peng L-N, Chen Y-T, Lin M-H, Liu L-K, and Chen L-K. Potentially inappropriate prescribing (IP) for elderly medical inpatients in Taiwan: A hospital-based study. Arch Gerontol Geriatr. 2012;55(1):148-51 https://doi.org/10.1016/j.archger.2011.07.001.
115. Lockery JE, Ernst ME, Broder JC, Orchard SG, Murray A, Nelson MR, et al. Prescription medication use in older adults without major cardiovascular disease enrolled in the Aspirin in Reducing Events in the Elderly (ASPREE) Clinical Trial. Pharmacotherapy. 2020;40(10):1042-53 https://doi.org/10.1002/phar.2461.
116. López NP, Villán YFV, Menéndez MIG, and Royuela yA. Potentially inappropriate prescribing in patients over 65 years-old in a primary care health centre. Aten Primaria. 2014;46(6):290-7 https://doi.org/10.1016/j.aprim.2013.12.007.
117. Lu J, Zhao W, Chen T, Xu Z, Sun X, Xie H, et al. Influence of guideline adherence and parameter control on the clinical outcomes in patients with diabetic nephropathy. BMJ Open Diabetes Res Care. 2020;8(1):e001166 https://doi.org/10.1136/bmjdrc-2019-001166.
118. Luz AC, de Oliveira MG, and Noblat L. Prescribing omissions among elderly Brazilian patients at their hospital admission and discharge: cross-sectional study. Int J Clin Pharm. 2018;40(6):1596-600 https://doi.org/10.1007/s11096-018-0726-5.
119. Maciulaitis R, Ozoliene D, Pilviniene R, and Preiksa R. Rational treatment of diabetes mellitus in Siauliai and Telsiai regions, Lithuania. Medicina. 2006;42(12):1013-9.
120. Maheshwari P and Shanmugarajan T. Evaluation of prescribing practices of metformin in patients with type-2 diabetes mellitus. Res J Pharm Technol. 2019;12(2):531-4 https://doi.org/10.5958/0974-360X.2019.00093.3.
121. Mahner M, Raus C, Ludwig F, Weckmann G, Stracke S, and Chenot J-F. Drug prescribing for patients with chronic kidney disease in general practice: A cross-sectional study. Dtsch Med Wochenschr. 2018;143(12):e99-e107 https://doi.org/10.1055/a-0584-1537.
122. Mahwi TO and Obied KA. Role of the pharmaceutical care in the management of patients with type 2 diabetes mellitus. Int J Pharm Sci Res. 2013;4(4):1363-9.
123. Maidana GM, Zullyone V, Samaniegoone L, Acostaone P, Mastroianni P, and Lugoone GB. Pharmaceutical interventions in patients with type 2 diabetes mellitus. Ars Pharm. 2017;58(1):21-8.
124. Manes C, Giacci L, Sciartilli A, D'Alleva A, and De Caterina R. Aspirin overprescription in primary cardiovascular prevention. Thromb Res. 2006;118(4):471-7 https://doi.org/10.1016/j.thromres.2005.09.013.
125. Manley HJ, McClaran ML, Overbay DK, Wright MA, Reid GM, Bender WL, et al. Factors associated with medication-related problems in ambulatory hemodialysis patients. Am J Kidney Dis. 2003;41(2):386-93 https://doi.org/10.1053/ajkd.2003.50048.

126. Margiani C, Zorcolo L, Mura P, Saba M, Restivo A, and Scintu F. Metformin-associated lactic acidosis and temporary ileostomy: A case report. J Med Case Reports. 2014;8(1):449 https://doi.org/10.1186/1752-1947-8-449.
127. Marroquín EC, Iglesia NM, and Cobos LP. Adequacy of medication in patients 65 years or older in teaching health centers in Cáceres, Spain. Rev Esp Salud Publica. 2012;86(4):419-34 https://doi.org/10.4321/S1135-57272012000400009.
128. Masoudi FA, Wang Y, Inzucchi SE, Setaro JF, Havranek EP, Foody JM, et al. Metformin and thiazolidinedione use in Medicare patients with heart failure. JAMA. 2003;290(1):81-5.
129. Matsumura Y, Yamaguchi T, Hasegawa H, Yoshihara K, Zhang Q, Mineno T, et al. Alert system for inappropriate prescriptions relating to patients' clinical condition. Methods Inf Med. 2009;48(06):566-73 https://doi.org/10.3414/ME9244.
130. McFarland MS, Cross LB, Gross B, Gentry C, Tunney J, and Patel U. Drug use evaluation of sitagliptin dosing by pharmacist versus nonpharmacist clinicians in an internal medicine department of a private physician-owned multispecialty clinic. J Manag Care Pharm. 2009;15(7):563-7.
131. Mechessa DF and Kebede B. Drug-related problems and their predictors among patients with diabetes attending the ambulatory clinic of Gebre Tsadik Shawo General Hospital, Southwest Ethiopia. Diabetes Metab Syndr Obes. 2020;13:3349-57.
132. Milligan F, Krentz A, and Sinclair A. Diabetes medication patient safety incident reports to the National Reporting and Learning Service: The care home setting. Diabet Med. 2011;28(12):1537-40 https://doi.org/10.1111/j.1464-5491.2011.03421.x.
133. Mino-León D, Galván-Plata ME, Anda-Garay JC, Noyola-García ME, and Cooper D. Inappropriate prescribing in older adults: Critical review of the literature and safety alerts. Rev Med Inst Mex Seguro Soc. 2018;56(S1):71-81.
134. Modesto ACF, Silveira EA, dos Santos Rodrigues AP, Lima DM, Provin MP, and Amaral RG. Prevalence of drug interaction in severely obese individuals and associated factors: Baseline results from a clinical trial. Sci Pharm. 2020;88(4):48-59 https://doi.org/10.3390/scipharm88040048.
135. Molist-Brunet N, Sevilla-Sánchez D, Puigoriol-Juvanteny E, González-Bueno J, Solà-Bonada N, Cruz-Grullón M, et al. Optimizing drug therapy in frail patients with type 2 diabetes mellitus. Aging Clin Exp Res. 2019:1-9 https://doi.org/10.1007/s40520-019-01342-z.
136. Mori AL, Carvalho RC, Aguiar PM, de Lima MG, Rossi MD, Carrillo JF, et al. Potentially inappropriate prescribing and associated factors in elderly patients at hospital discharge in Brazil: A cross-sectional study. Int J Clin Pharm. 2017;39(2):386-93 https://doi.org/10.1007/s11096-017-0433-7.
137. Muller C, Dimitrov Y, Imhoff O, Richter S, Ott J, Krummel T, et al. Oral antidiabetics use among diabetic type 2 patients with chronic kidney disease. Do nephrologists take account of recommendations? J Diabetes Complications. 2016;30(4):675-80 https://doi.org/10.1016/j.jdiacomp.2016.01.016.
138. Mwita JC, Godman B, and Esterhuizen TM. Statin prescription among patients with type 2 diabetes in Botswana: Findings and implications. BMC Endocr Disord. 2020;20(36):1-9 https://doi.org/https://doi.org/10.1186/s12902-020-0516-7.
139. Najim HS, Sulaiman SA, and Khan TM. Metformin use with contraindications in diabetic type-2 patients. HEALTHMED. 2010;4(4):759-64.
140. Nelson E and Saeed M. Adherence with the renal licences of antidiabetic medications in diabetes outpatient clinics at a large university teaching hospital. in Abstracts of the Diabetes UK Professional Conference. 2018.
141. Niehoff KM, Rajeevan N, Charpentier PA, Miller PL, Goldstein MK, and Fried TR. Development of the tool to reduce inappropriate medications (TRIM): A clinical decision

support system to improve medication prescribing for older adults. Pharmacotherapy. 2016;36(6):694-701 https://doi.org/10.1002/phar.1751.

142. Nuñez-Montenegro A, Montiel-Luque A, Martin-Aurioles E, Garcia-Dillana F, Krag-Jiménez M, and González-Correa JA. Evaluation of inappropriate prescribing in patients older than 65 years in primary health care. J Clin Med. 2019;8:305-13 https://doi.org/10.3390/jcm8030305.

143. Ogamba OE, Potential drug-drug interactions among patients with type 2 diabetes and hypertension in Kissi Teaching and Refferal Hospital, Kenya. 2016, University of Nairobi.

144. Oktora MP, Alfian SD, Bos HJ, Schuiling-Veninga CCM, Taxis K, Hak E, et al. Trends in polypharmacy and potentially inappropriate medication (PIM) in older and middle-aged people treated for diabetes. Br J Clin Pharmacol. 2020:1-11 https://doi.org/10.1111/bcp.14685.

145. Pasina L, Novella A, Elli C, Nobili A, and Ianes A. Inappropriate use of antiplatelet agents for primary prevention in nursing homes: An Italian multicenter observational study. Geriatr Gerontol Int. 2020:1-5 https://doi.org/10.1111/ggi.13984.

146. Patel PA, Liang L, Khazanie P, Hammill BG, Fonarow GC, Yancy CW, et al. Antihyperglycemic medication use among Medicare beneficiaries with heart failure, diabetes mellitus, and chronic kidney disease. Circ Heart Fail. 2016;9(7):e002638 https://doi.org/10.1161/circheartfailure.115.002638.

147. Penfornis A, Blicklé JF, Fiquet B, Quéré S, and Dejager S. How are patients with type 2 diabetes and renal disease monitored and managed? Insights from the observational OREDIA study. Vasc Health Risk Manag. 2014;10:341-52 https://doi.org/10.2147/VHRM.S60312.

148. Pitkala KH, Strandberg TE, and Tilvis RS. Inappropriate drug prescribing in home-dwelling, elderly patients: A population-based survey. Arch Intern Med. 2002;162(15):1707-12.

149. Pongwecharak J, Tengmeesri N, Malanusorn N, Panthong M, and Pawangkapin N. Prescribing metformin in type 2 diabetes with a contraindication: Prevalence and outcome. Pharm World Sci. 2009;31(4):481-6 https://doi.org/10.1007/s11096-009-9303-2.

150. Prado MAMBd, Francisco PMSB, and Barros MBdA. Diabetes in the elderly: Drug use and the risk of drug interaction. Cien Saude Colet. 2016;21(11):3447-58 https://doi.org/DOI: 10.1590/1413-812320152111.24462015.

151. Ramachandran M, Loi B, Ariff NM, Chuan NO, Sham SYZ, Thambiah SC, et al. Appropriateness of metformin prescription for type 2 diabetes mellitus patients with chronic kidney disease (Stages 3-5). Malays J Pathol. 2020;42(1):71-6.

152. Rigler SK, Jachna CM, Perera S, Shireman TI, and Eng ML. Patterns of potentially inappropriate medication use across three cohorts of older Medicaid recipients. Ann Pharmacother. 2005;39(7):1175-81 https://doi.org/10.1345/aph.1E581.

153. Romley JA, Gong C, Jena AB, Goldman DP, Williams B, and Peters A. Association between use of warfarin with common sulfonylureas and serious hypoglycemic events: retrospective cohort analysis. BMJ. 2015;351:h6223: https://doi.org/10.1136/bmj.h6223.

154. Ruiz-Tamayo I, Franch-Nadal J, Mata-Cases M, Mauricio D, Cos X, Rodriguez-Poncelas A, et al. Noninsulin antidiabetic drugs for patients with type 2 diabetes mellitus: Are we respecting their contraindications? J Diabetes Res. 2016;2016 https://doi.org/10.1155/2016/7502489.

155. Salgueiro E, Elizarde BC, Elola AI, García-Pulido B, Nicieza-García ML, and Manso G. The most common STOPP/START criteria in Spain. A review of the literature. Rev Esp Geriatr Gerontol. 2018;53(5):274-8 https://doi.org/10.1016/j.regg.2018.03.001.

156. Samardzic I and Bacic-Vrca V. Incidence of potential drug-drug interactions with antidiabetic drugs. Pharmazie. 2015;70(6):410-5 https://doi.org/10.1691/ph.2015.4777.

157. Sankar V, Saaed Y, Joseph R, Azizi H, and Thomas P. Serious drug-drug interactions in the prescriptions of diabetic patients. Medical Sciences. 2015;3(4):93-103 https://doi.org/10.3390/medsci3040093.
158. Sato I, Yamamoto Y, Kato G, and Kawakami K. Potentially inappropriate medication prescribing and risk of unplanned hospitalization among the elderly: A self-matched, case-crossover study. Drug Saf. 2018;41(10):959-68 https://doi.org/10.1007/s40264-018-0676-9.
159. Schindler E, Hohmann C, and Culmsee C. Medication review by community pharmacists for type 2 diabetes patients in routine care: Results of the DIATHEM-study. Front Pharmacol. 2020;11:1176: https://doi.org/10.3389/fphar.2020.01176.
160. Scotton DW, Wierman H, Coughlan A, Walters M, and Kuhn C. Assessing the appropriate use of metformin in an inpatient setting and the effectiveness of two pharmacy-based measures to improve guideline adherence. Qual Manag Health Care. 2009;18(1):71-6.
161. Secoli S-R, Figueras A, Lebrao ML, de Lima FD, and Santos JLF. Risk of potential drug-drug interactions among Brazilian elderly. Drugs Aging. 2010;27(9):759-70.
162. Shah A, Naqvi AA, and Ahmad R. The need for providing pharmaceutical care in geriatrics: A case study of diagnostic errors leading to medication-related problems in a patient treatment plan. Arch Pharm Pract. 2016;7(3):87-94 https://doi.org/10.4103/2045-080X.186173.
163. Shareef J, Fernandes J, and Samaga L. Clinical pharmacist interventions in drug therapy in patients with diabetes mellitus and hypertension in a university teaching hospital. Int J Pharm Sci Res. 2015;6(10):4424-32 https://doi.org/10.13040/IJPSR.0975-8232.6(10).4424-32.
164. Shareef J, Fernandes J, and Samaga L. Assessment of clinical pharmacist interventions in drug therapy in patients with diabetes mellitus in a tertiary care teaching hospital. Diabetes Metab Syndr. 2016;10(2):82-7 https://doi.org/10.1016/j.dsx.2015.09.017.
165. Sharma R, Chhabra M, Vidyasagar K, Rashid M, Fialova D, and Bhagavathula AS. Potentially inappropriate medication use in older hospitalized patients with type 2 diabetes: A cross-sectional study. Pharmacy. 2020;8:219: https://doi.org/10.3390/pharmacy8040219.
166. Silvestre J, Carvalho S, Mendes V, Coelho L, Tapadinhas C, Ferreira P, et al. Metformin-induced lactic acidosis: A case series. J Med Case Reports. 2007;1:176: https://doi.org/10.1186/1752-1947-1-126.
167. Simons LA and Chung E. Are high coronary risk patients missing out on lipid lowering drugs in Australia? Med J Aust. 2014;201(4):213-6 https://doi.org/10.5694/mja14.00249.
168. Siripala U, Premadasa S, Samaranayake N, and Wanigatunge C. Usefulness of STOPP/START criteria to assess appropriateness of medicines prescribed to older adults in a resource-limited setting. Int J Clin Pharm. 2019;41(2):525-530 https://doi.org/10.1007/s11096-019-00786-7.
169. Soorapan S, Assessing pharmacist's intervention in supporting the management of type 2 diabetes in a primary care setting. 2002, Robert Gordon University: Aberdeen.
170. Spanopoulos D, Barrett B, Busse M, Roman T, and Poole C. Prescription of DPP-4 inhibitors to type 2 diabetes mellitus patients with renal impairment: A UK primary care experience. Clin Ther. 2018;40(1):152-4 https://doi.org/10.1016/j.clinthera.2017.11.009.
171. Stewart AL, Snodgrass J, Schontz M, and Parekh M. A student pharmacist-led medication reconciliation service and its impact on the identification of drug-related problems in an ambulatory clinic. Curr Pharm Teach Learn. 2015;7(5):575-83 https://doi.org/10.1016/j.cptl.2015.06.020.
172. Sulkin TV, Bosman D, and Krentz AJ. Contraindications to metformin therapy in patients with NIDDM. Diabetes Care. 1997;20(6):925-8 https://doi.org/10.2337/diacare.20.6.925.

173. Swanoski MT, Little MM, St Hill CA, Ware KB, Chapman S, and Lutfiyya MN. Potentially inappropriate medication prescribing in US older adults with selected chronic conditions. Consult Pharm. 2017;32(9):525-34 https://doi.org/10.4140/TCP.n.2017.525.
174. Sweileh WM. Contraindications to metformin therapy among patients with type 2 diabetes mellitus. Pharm World Sci. 2007;29(6):587-92 https://doi.org/10.1007/s11096-007-9095-1.
175. Tahir MT and Amiruddin R. Implementation of pharmaceutical care in resolving drug problems in type 2 diabetes mellitus. Folia Medica Indonesiana. 2015;51(2):80-5.
176. Taner N, Çattık BN, and Berk B. A contraindication to metformin therapy: renal impairment-adherence to prescribing guidelines at a hospital in Turkey. Acta Pharm Sci. 2018;56(1):63-70 https://doi.org/10.23893/1307-2080.APS.05604.
177. Terán-Álvarez L, González-García M, Rivero-Pérez A, Alonso-Lorenzo J, and Tarrazo-Suárez J. Potentially inappropriate prescription according to the " STOPP" criteria in heavily polymedicated elderly patients. Semergen. 2016;42(1):1-9 https://doi.org/10.1016/j.semerg.2014.10.018.
178. Tirkkonen T, Heikkilä P, Huupponen R, and Laine K. Potential CYP2C9-mediated drug–drug interactions in hospitalized type 2 diabetes mellitus patients treated with the sulphonylureas glibenclamide, glimepiride or glipizide. J Intern Med. 2010;268(4):359-66 https://doi.org/10.1111/j.1365-2796.2010.02257.x.
179. Toth EL, Majumdar SR, Guirguis LM, Lewanczuk RZ, Lee TK, and Johnson JA. Compliance with clinical practice guidelines for type 2 diabetes in rural patients: Treatment gaps and opportunities for improvement. Pharmacotherapy. 2003;23(5):659-65 https://doi.org/10.1592/phco.23.5.659.32203.
180. Vaccaro O, Boemi M, Cavalot F, De Feo P, Miccoli R, Patti L, et al. The clinical reality of guidelines for primary prevention of cardiovascular disease in type 2 diabetes in Italy. Atherosclerosis. 2008;198(2):396-402 https://doi.org/10.1016/j.atherosclerosis.2007.10.026.
181. van Roozendaal BW and Krass I. Development of an evidence-based checklist for the detection of drug related problems in type 2 diabetes. Pharm World Sci. 2009;31(5):580-95 https://doi.org/10.1007/s11096-009-9312-1.
182. Wen YW, Tsai YW, Huang WF, Hsiao FY, and Chen PF. The potentially inappropriate prescription of new drug: Thiazolidinediones for patients with type II diabetes in Taiwan. Pharmacoepidemiol Drug Saf. 2011;20(1):20-9 https://doi.org/10.1002/pds.2010.
183. Wermeille J, Bennie M, Brown I, and McKnight J. Pharmaceutical care model for patients with type 2 diabetes: integration of the community pharmacist into the diabetes team–a pilot study. Pharm World Sci. 2004;26(1):18-25 https://doi.org/10.1023/B:PHAR.0000013465.24857.a8.
184. Wood D, Plehwe W, and Colman P. Aspirin usage in a large teaching hospital diabetes clinic setting. Diabet Med. 1999;16(7):605-8 https://doi.org/10.1046/j.1464-5491.1999.00101.x.
185. Woodward A, Bayley D, Overend L, and Gill G. Antiplatelet drug use in a diabetic clinic. QJM-Int J Med. 2007;100(9):547-50 https://doi.org/10.1093/qjmed/hcm058.
186. Xin C, Ge X, Zheng L, and Huang P. Evaluation of pharmaceutical care in a diabetes ward from China: A pre-and post-intervention study. Int J Clin Pharm. 2016;38(1):34-40 https://doi.org/10.1007/s11096-013-9855-z.
187. Yeste-Gomez I, Duran-Garcia ME, Muino-Miguez A, Gomez-Antunez M, Lopez-Berastegui O, and Sanjurjo-Saez M. Potentially inappropriate prescriptions in the ambulatory treatment of elderly patients. Rev Calid Asist. 2014;29(1):22-8 https://doi.org/10.1016/j.cali.2013.08.001.
188. Yimama M, Jarso H, and Desse TA. Determinants of drug-related problems among ambulatory type 2 diabetes patients with hypertension comorbidity in South West Ethiopia: a prospective cross sectional study. BMC Res Notes. 2018;11:679: https://doi.org/10.1186/s13104-018-3785-8.

189. Zanatta L, Cort FND, Mathias NS, and Argenta C. Analysis of drug interactions and epidemiological profile of individuals with diabetes mellitus in primary care. Rev. Enferm. UFSM. 2020;10(e47):1-19 https://doi.org/10.5902/2179769240175.
190. Zazuli Z, Rohaya A, and Adnyana IK. Drug-related problems in Type 2 diabetic patients with hypertension in Cimahi, West Java, Indonesia: A prospective study. Int J Green Pharm. 2017;11(2):s298-s304.
